# Two Independent Plastid *accD* Transfers to the Nuclear Genome of *Gnetum* and Other Insights on Acetyl-CoA Carboxylase Evolution in Gymnosperms

**DOI:** 10.1093/gbe/evz059

**Published:** 2019-03-29

**Authors:** Edi Sudianto, Shu-Miaw Chaw

**Affiliations:** 1 Biodiversity Program, Taiwan International Graduate Program, Academia Sinica and National Taiwan Normal University, Taipei, Taiwan; 2 Department of Life Science, National Taiwan Normal University, Taipei, Taiwan; 3 Biodiversity Research Center, Academia Sinica, Taipei, Taiwan

**Keywords:** accD, acetyl-CoA carboxylase (ACCase), fatty acid biosynthesis, plastid-to-nucleus gene transfer, plastid localization, evolution

## Abstract

Acetyl-CoA carboxylase (ACCase) is the key regulator of fatty acid biosynthesis. In most plants, ACCase exists in two locations (cytosol and plastids) and in two forms (homomeric and heteromeric). Heteromeric ACCase comprises four subunits, three of them (ACCA–C) are nuclear encoded (nr) and the fourth (ACCD) is usually plastid encoded. Homomeric ACCase is encoded by a single nr-gene (*ACC*). We investigated the ACCase gene evolution in gymnosperms by examining the transcriptomes of newly sequenced *Gnetum ula*, combined with 75 transcriptomes and 110 plastomes of other gymnosperms. *AccD-*coding sequences are elongated through the insertion of repetitive DNA in four out of five cupressophyte families (except Sciadopityaceae) and were functionally transferred to the nucleus of gnetophytes and *Sciadopitys*. We discovered that, among the three genera of gnetophytes, only *Gnetum* has two copies of nr-*accD*. Furthermore, using protoplast transient expression assays, we experimentally verified that the nr-*accD* precursor proteins in *Gnetum* and *Sciadopitys* can be delivered to the plastids. Of the two nr-*accD* copies of *Gnetum*, one dually targets plastids and mitochondria, whereas the other potentially targets plastoglobuli. The distinct transit peptides, gene architectures, and flanking sequences between the two *Gnetum accD*s suggest that they have independent origins. Our findings are the first account of two distinctly targeted nr-*accD*s of any green plants and the most comprehensive analyses of ACCase evolution in gymnosperms to date.

## Introduction

Fatty acid biosynthesis in plants begins with the conversion of acetyl-CoA into malonyl-CoA, catalyzed by acetyl-CoA carboxylase (ACCase). Malonyl-CoA is an essential substrate for fatty acid formation (see reviews by Brown et al. [2010] and Huerlimann and Heimann [2013]). There are generally two types of ACCases in plants: the multisubunit heteromeric ACCase (ACCA–D) in plastids and the single-polypeptide homomeric ACCase (ACC) in the cytosol (Sasaki and Nagano 2004). The plastid heteromeric ACCase is similar to the prokaryotic ACCase and believed to have originated from cyanobacteria, whereas the cytosol homomeric ACCase is the eukaryotic type ACCase (Nikolau et al. 2003). Heteromeric ACCase consists of four subunits: 1) the alpha-subunit of carboxyltransferase (α-CT; encoded by *accA*), 2) biotin-carboxyl carrier protein (BCCP; encoded by *accB*), 3) biotin-carboxylase (BC; encoded by *accC*), and 4) the beta-subunit of carboxyltransferase (β-CT; encoded by *accD*). Genes encoding the former three reside in the nuclei, whereas *accD* resides in the plastids of most plant species. Loss of plastid *accD* has been reported in some seed plants, including monocots: Poaceae (Konishi and Sasaki 1994) and *Acorus* (Goremykin et al. 2005); eudicots: *Trifolium* (Magee et al. 2010), some *Silene* species (Sloan et al. 2012), Campanulaceae (Rousseau-Gueutin et al. 2013), and a few species of *Pelargonium* (Röschenbleck et al. 2017); and gymnosperms: gnetophytes (Wu et al. 2009) and *Sciadopitys* (Hsu, Wu, and Chaw 2016; Li et al. 2016). Homomeric ACCase is encoded by a single gene, *ACC* (Nikolau et al. 2003).

Although both plastid and cytosol ACCases convert acetyl-CoA into malonyl-CoA, the two compartments produce distinct end products. Malonyl-CoA is mainly converted into free fatty acids in the plastids (Brown et al. 2010), whereas in the cytosol it is used to synthesize flavonoids, anthocyanins, very long-chain fatty acids, and for the malonylation of D-amino acids and ethylene precursors (Sasaki and Nagano 2004). Thus, both ACCases play vital roles in plants. The loss of either of these genes is lethal, as was demonstrated in *Arabidopsis* (Baud et al. 2003) and tobacco (Kode et al. 2005). The only exceptions were reported in grasses (Poaceae) and *Silene noctiflora*, in which heteromeric ACCase is absent (supplementary fig. S1, Supplementary Material online; Konishi et al. 1996; Rockenbach et al. 2016), but a copy of homomeric ACCase has acquired plastid-targeting transit peptides (TPs) and replaced the function of heteromeric ACCase in plastids (Gornicki et al. 1997; Podkowinski et al. 2003; Rockenbach et al. 2016). In other cases (supplementary fig. S1, Supplementary Material online), both heteromeric and homomeric ACCase coexist in the plastids of *Arabidopsis* (Babiychuk et al. 2011) and Geraniaceae (Park et al. 2017).

Little is known about ACCase evolution in gymnosperms. Extant gymnosperms encompass about 1,100 species in 83 genera and 12 families (Christenhusz and Byng 2016). They comprise five groups—ginkgo, cycads, gnetophytes, Pinaceae (conifers I), and cupressophytes (conifers II) (Chaw et al. 2000). The cupressophytes are made up of five families—Cupressaceae, Taxaceae, Sciadopityaceae, Araucariaceae, and Podocarpaceae (Gernandt et al. 2011).

To date, the only characterization of ACCase genes in gymnosperms is limited to the loss of plastid-encoded *accD* from gnetophytes (Wu et al. 2009) and *Sciadopitys* (Hsu, Wu, and Chaw 2016; Li et al. 2016). Whether the lost plastid *accD* has been transferred to the nuclear genomes of these two lineages has not been verified. As a previous study identified a partial ACCD transcript in *Sciadopitys* (Li et al. 2016), we hypothesized that its *accD* was transferred to the nucleus. However, no information is available for the gnetophytes; it is unknown whether they retained heteromeric ACCase or if it was replaced with homomeric ACCase (as in grasses). In addition, the plastid *accD* sequences of cupressophyte genera (Hirao et al. 2008; Yi et al. 2013; Li et al. 2018) and a Pinaceae genus, *Tsuga* (Sudianto et al. 2016), are much longer than their homologs from other gymnosperms. This elongation may have accelerated *accD* nucleotide substitution rates in both *Tsuga* (Sudianto et al. 2016) and cupressophytes (Li et al. 2018). Whether the accelerated rates of ACCD subunit in these taxa influence the other ACCase subunits remains uncertain.

Here, we report the characterization of genes and transcripts encoding heteromeric and homomeric ACCases in *Gnetum ula* and 75 additional gymnosperm transcriptomes sampled from public repository data. We also assess if both ACCase forms are present in gymnosperms. Furthermore, we investigate the evolutionary fate of *accD* across the five major groups of gymnosperms, including the *accD* that was lost from gnetophytes and *Sciadopitys* plastomes. Finally, we perform a protoplast transient expression assay to identify the localization target of nuclear-encoded *accD* from gnetophytes and *Sciadopitys*.

## Materials and Methods

### Isolation of Nucleic Acids and cDNA Synthesis

We collected *G.ula* (voucher Chaw 1569) and *Sciadopitys verticillata* (voucher Chaw 1496) from the living plants in the green house of Academia Sinica and Floriculture Experimenter Center, Taipei, respectively. Both specimens were deposited in the Herbarium of Academia Sinica, Taipei (HAST). Total RNA was extracted from both tissues following the protocol of Hsu, Wu, and Chaw (2016). First strand cDNA was synthesized using RevertAid H Minus First Strand cDNA Synthesis Kit (Thermo Fischer Scientific, Waltham) using random hexamer primers.

### Sequence Retrieval

Coding regions of the plastid *accD* gene, including three nonvascular plants and 116 species of gymnosperms, were collected from GenBank and transcriptome data (supplementary table S1, Supplementary Material online). For the 76 transcriptomes analyzed in this study, we downloaded 74 assembled gymnosperm transcriptomes from oneKP (Matasci et al. 2014) and NCBI TSA databases (https://www.ncbi.nlm.nih.gov/genbank/tsa/; Last accessed on 10 November 2018), including 54 cupressophytes, 11 Pinaceae, 4 gnetophytes, 4 cycads, and 1 ginkgo (supplementary table S2, Supplementary Material online). We *de novo* assembled the transcriptomes of the two remaining *Gnetum* species—*G. parvifolium* and *G. ula*. *Gnetumparvifolium* reads were obtained from NCBI SRA (SRX1133345).

### RNA Sequencing, Transcriptome Assembly, and Identification of ACCase Transcripts

RNA-Seq of *G. ula* was sequenced using an Illumina HiSeq2500 platform, yielding ∼100 million paired-end reads (2× 100-bp length). Trimmomatic (Bolger et al. 2014) was used to remove low-quality reads and adapters from both the newly sequenced *G. ula* and retrieved *G. parvifolium* reads. Transcriptomes of both species were de novo assembled using SOAPdenovo-Trans 1.03 (Xie et al. 2014). TransDecoder 4.0.0 (Haas et al. 2013) was used to identify candidate coding regions. We used BlastP (*E*-value <1e^−5^) to identify the potential ACCase proteins from the gymnosperm transcriptome assemblies. Protein sequences of four heteromeric ACCase (*accA*, *accB*, *accC*, and *accD*) and two homologs of homomeric ACCase (*ACC*1 and *ACC*2) from *Arabidopsis* were used as queries.

### Tandem Repeats and TP Identification

Tandem repeats (TRs) of the elongated ACCD in cupressophytes were identified using T-REKS (Jorda and Kajava 2009) with default parameters. TP sequences were predicted using a number of prediction tools, including TargetP (Emanuelsson et al. 2007), Predotar (Small et al. 2004), ProteinProwler (Hawkins and Bodén 2006), and LOCALIZER (Sperschneider et al. 2017).

### Sequence Alignment and Phylogenetic Tree Reconstruction

ACCase protein sequences were aligned using MAFFT v7 (Katoh and Standley 2013) in Auto setting. Sequences with <50% total protein length and duplicated sequences with 100% similarity were removed from further analyses. ProtTest3 (Darriba et al. 2011) was used to find the best-fit model for phylogenetic tree reconstruction. Maximum likelihood (ML) trees were reconstructed using RAxML 8.2.10 (Stamatakis 2014) in the recommended JTTGAMMAI model with 1,000 bootstrap replications. Tajima’s relative rate tests were performed using MEGA 7 (Kumar et al. 2016).

### Identification of ACCase Genes in Nuclear Genomes

BlastN was used to search for ACCase genes in the nuclear genomes of *Ginkgo biloba* (Guan et al. 2016; http://gigadb.org/dataset/100209; Last accessed on 3 January 2018), *Pinus taeda* (Zimin et al. 2017; http://pinegenome.org/pinerefseq; Last accessed on 4 January 2018), and draft *G. ula* using their respective ACCase transcripts as the queries. Splign (Kapustin et al. 2008) and Easyfig (Sullivan et al. 2011) were used to identify exon–intron boundaries and illustrate the gene structures, respectively.

### Protoplast Transient Expression of nr-ACCDs TPs

The putative TP sequences from *G. ula* (both nr-*accD*1 and nr-*accD*2) and *Sciadopitys* were amplified from cDNA of respective species with specific primers (see supplementary table S3, Supplementary Material online, for primers used). Amplified fragments were each cloned into the p326-GFP vector at *Xba*I and *Bam*HI restriction sites. These constructs were transfected into *Arabidopsis* protoplasts following polyethylene glycol-mediated transformation described by Lee and Hwang (2011). In brief, *Arabidopsis* protoplasts were isolated from the leaves of 3-week-old plants as previously described (Wu et al. 2009) and then transformed with 10–20 µg of plasmid DNA from three GFP constructs described above. Plasmid DNA from p326-GFP vector was also used as a control to specify cytosol localization. Protoplasts and plasmids were incubated in the dark for 16 h at room temperature. The transformed protoplast images were captured using a Zeiss LSM780 ELYRA PS1 confocal microscope system.

### Molecular Dating and Estimation of Absolute Nucleotide Substitution Rates

We sampled 34 gymnosperm genera in which all 5 ACCase sequences were available from the plastome and transcriptome data. Both synonymous (*d*_S_) and nonsynonymous (*d*_N_) substitution rates of these ACCase subunits were estimated using PAMLX (Xu and Yang 2013). We used the program CODEML with the following parameters: runmode = 0, seqtype = 1, CodonFreq = 2, estFreq = 0, model = 1, and cleandata = 1. We concatenated the *accA–C* sequences to calculate the *d*_S_ and *d*_N_ of nr-heteromeric ACCase. The constraint tree topology (supplementary fig. S2, Supplementary Material online) was reconstructed using 29 plastid-encoded photosynthetic genes. The relative divergence times were estimated using RelTime (Tamura et al. 2012) in MEGA 7.0. Seven estimated points from TimeTree (Kumar et al. 2017) were used as the calibration points (supplementary fig. S3, Supplementary Material online). The absolute synonymous (*R*_S_) and nonsynonymous substitution rates (*R*_N_) were derived by dividing the *d*_N_ and *d*_S_ branch lengths by their respective divergence times. We compared the absolute branch lengths of 1) *accD* versus *ACC*, 2) *accD* versus concatenated *accA–C* (nr-heteromeric ACCase), and 3) nr-heteromeric ACCase versus *ACC* to determine their correlations. Only terminal branches were considered in our analyses.

## Results

### Gymnosperms Have Both Heteromeric and Homomeric ACCases

**Fig. 1.**
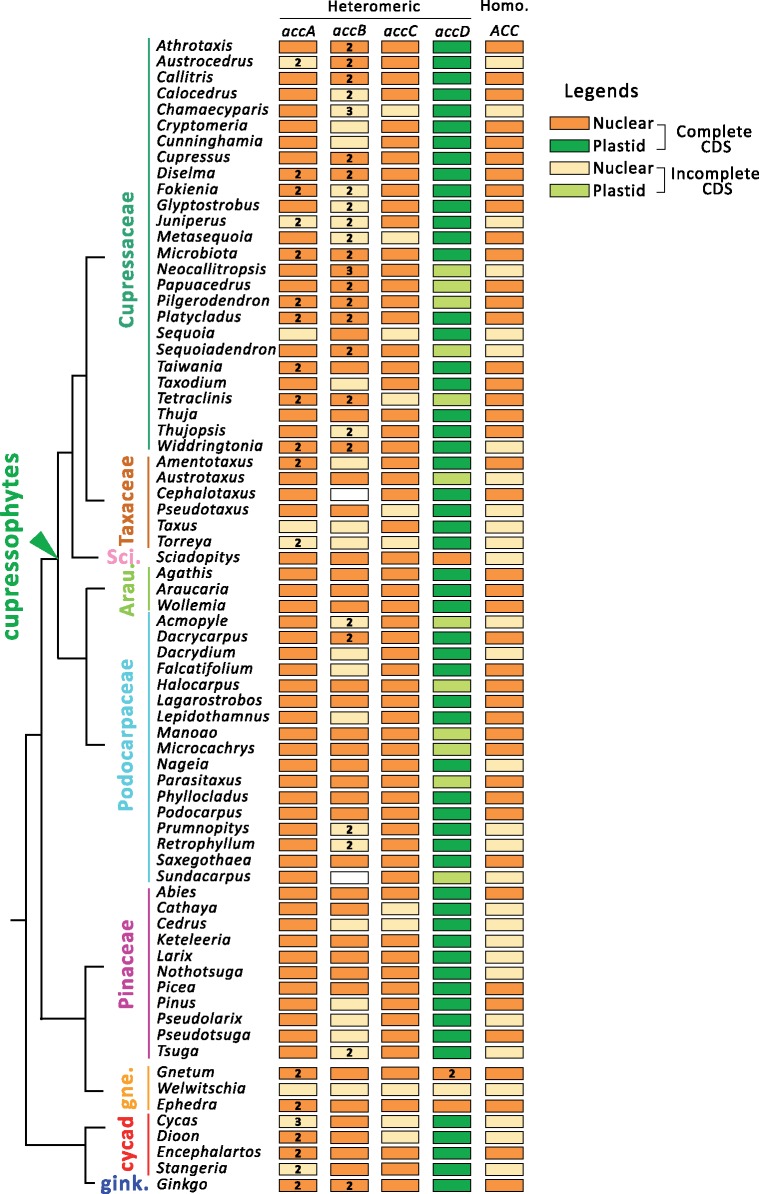
—The gymnosperms contain both heteromeric and homomeric ACCase transcripts. The ACCase complex is differentiated into two groups: heteromeric (including *accA*, *accB*, *accC*, and *accD*) and homomeric (*ACC*). The color-filled boxes indicate the location and completeness of the transcript. The number inside the boxes indicates the presence of two or three transcript copies in some species. Phylogenetic relationships between the groups were derived from Li et al. (2017). Sci., Sciadopityaceae; gne., gnetophytes; and gink., ginkgo.

Transcripts encoding heteromeric and homomeric ACCases were identified in most gymnosperms. Transcripts of *accA–D* were present in all examined species, except *Cephalotaxus* and *Sundacarpus*, whose *accB* transcript was absent, possibly due to fragmented transcriptome assemblies (fig. 1 ). All *accA*, *accB*, and *accC* transcripts carried plastid-targeting TPs (supplementary table S4, Supplementary Material online). Two to three unique transcripts of *accA* and *accB* were detected in ginkgo, cycads, gnetophytes, and cupressophytes (fig. 1). In Cupressaceae, some duplicated *accA* and *accB* transcripts contained mitochondria-targeting TPs (supplementary table S4, Supplementary Material online). No duplicated *accC* transcript was detected, suggesting that there is only a single copy of *accC* in gymnosperms. We detected two copies of *accD* transcripts in *Gnetum* and one copy in *Ephedra*, *Welwitschia*, and *Sciadopitys*, though this gene is absent from their plastid genomes (plastomes). A single copy of homomeric ACCase (*ACC*) transcripts was present in all examined gymnosperms, and no gymnosperms had plastid-targeting TPs (supplementary table S4, Supplementary Material online).

### TR Insertions Influence ACCD Length in Gymnosperms

**Fig. 2.**
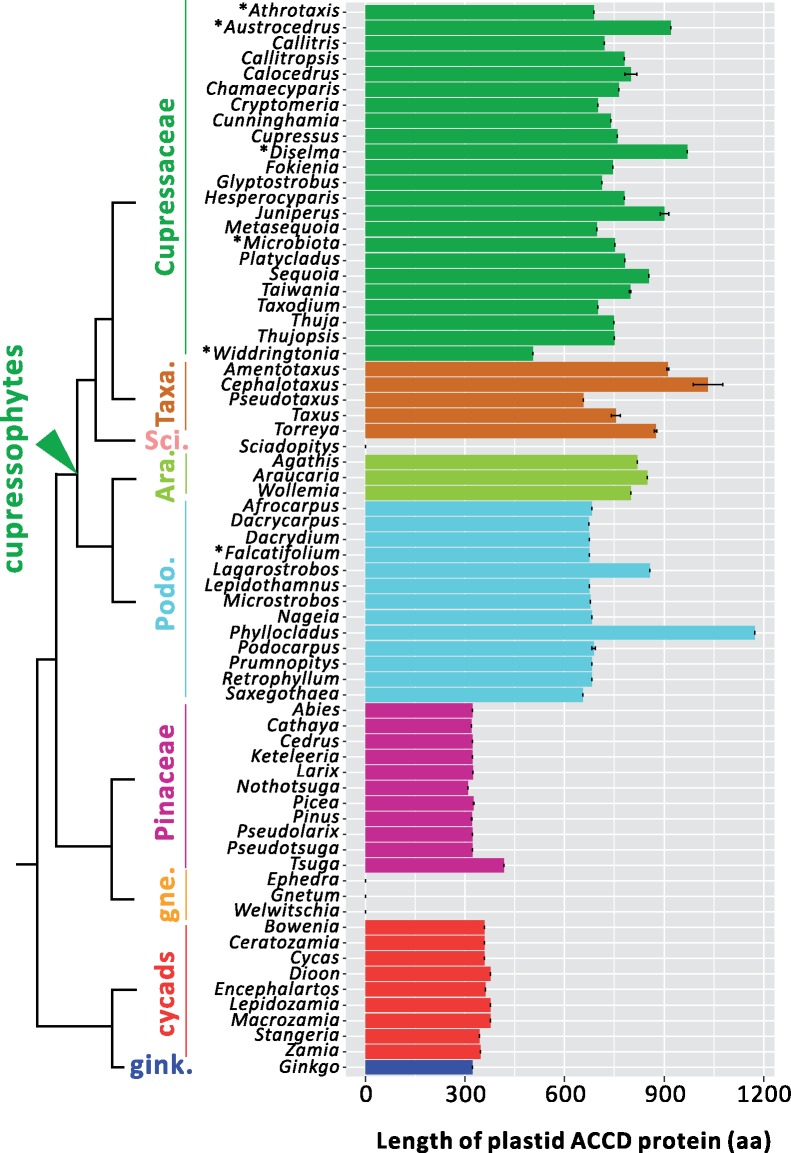
—Comparisons of plastid ACCD among the gymnosperms. The lengths of ACCD amino acid sequences were deduced from the plastid genes or transcriptomes of gymnosperms. Each group is coded by a specific color, as depicted in the legend. *AccD* is absent from the plastomes of *Sciadopitys* and gnetophytes. See supplementary table S2, Supplementary Material online, for a complete list of sampled species and their accession numbers. Asterisks (*) denote the sequences that were derived only from transcriptomes (i.e., no plastome available). Phylogenetic relationships between the groups were derived from Li et al. (2017). Taxa., Taxaceae; Sci., Sciadopityaceae; Ara., Araucariaceae; Podo., Podocarpaceae; gne., gnetophytes; and gink., ginkgo.

ACCDs in gymnosperms vary in length from 309 to 1,173 amino acids (aa) (fig. 2 and supplementary table S1, Supplementary Material online). Cupressophytes have significantly longer ACCDs than other gymnosperms (Mann–Whitney test, *P *<* *0.001). In cupressophytes, *Widdringtonia* (504 aa) has the shortest ACCD sequence, whereas the ACCD sequence of *Phyllocladus* (1,173 aa) is ∼3–4-fold longer than the ACCD sequences of three other major groups: the Pinaceae, ginkgos, and cycads (ranging from 300 to 400 aa). Other Podocarpaceous genera have ACCD sequences 1.3–2-fold shorter than *Phyllocladus*’ (670–857 aa).

All sampled cupressophyte ACCDs bear insertions with little to no similarities to those of Pinaceae, ginkgo, and cycads (supplementary fig. S4, Supplementary Material online). These insertions contain various types of TRs that differ among closely related lineages. The number of TR types ranges from one to six for each cupressophyte genus. For example, 13 genera in the Araucariales order (including Araucariaceae and Podocarpaceae) share one TR type (see red font in table 1). Other than order-specific TRs, family-specific TRs were also identified in Araucariaceae (3 TRs, blue), Podocarpaceae (1 TR, purple), and Cupressaceae (1 TR, green). In addition, genera of the subfamily Cupressoideae share one subfamily-specific TR (brown). No common TR type was found in Taxaceae.

**Table 1:** Tandem Repeat Units Identified in the Plastid *accD* Protein of 44 Sampled Cupressophytes Genera**^a^**

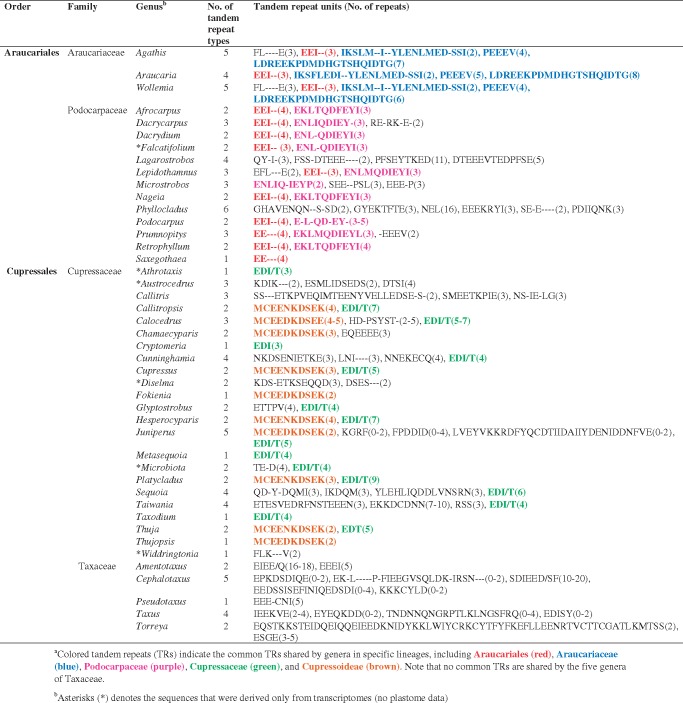

### The Plastid *accD*s of Gnetophytes and *Sciadopitys* Were Transferred to the Nucleus and *Gnetum* Retains Two Copies of nr-ACCD

**Fig. 3.**
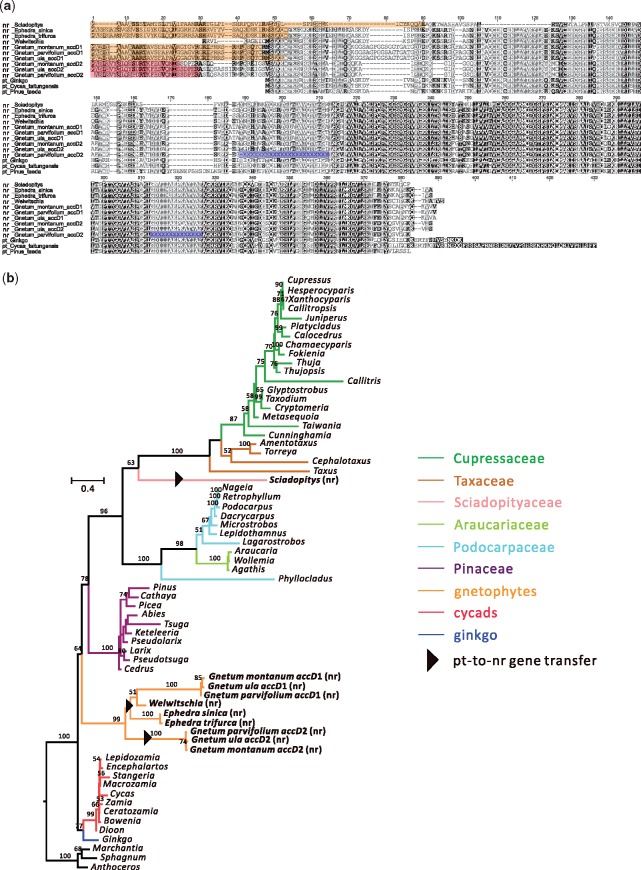
—Sequence comparisons among the ACCD of *Sciadopitys*, gnetophytes, and other gymnosperm representatives. (*a*) Identification of predicted nr-ACCD proteins from *Sciadopitys* and three genera of gnetophytes. The putative nr-ACCDs were aligned with the plastid (pt) ACCDs of three other gymnosperms: *Ginkgo*, *Cycas*, and *Pinus*. Orange shading denotes the TP sequences as predicted by LOCALIZER and TargetP. Red shading denotes the TPs predicted by TargetP only. The gaps in the *Gnetum parvifolium* transcriptome assembly are highlighted with blue shading. (*b*) ML tree of ACCD sequences. The nr-ACCD sequences of *Sciadopitys* and gnetophytes (bold) were aligned with the plastid ACCD sequences of other 53 sampled gymnosperm species. Bootstrapping supports for the node are shown when they are >50%. A black arrow indicates a plastid-to-nuclear *accD* gene transfer event. *Marchantia*, *Sphagnum*, and *Anthoceros* were designated as the outgroups.

We detected ACCD transcripts in the transcriptome assemblies of gnetophytes and *Sciadopitys*, with predicted protein sequences ranging from 312 aa (*Sciadopitys*) to 370 aa (*Gnetum*) despite the absence of *accD* from their plastomes. This finding suggests that *accD* was transferred to the nuclear genomes of both lineages. The nuclear-encoded (nr) ACCD of *Sciadopitys* discovered in this study is 100 aa longer than previously reported by Li et al. (2016). We identified two homologous ACCD sequences in the three *Gnetum* transcriptomes (*G. ula*, *G. montanum*, and *G. parvifolium*; fig. 3). By contrast, the two *Ephedra* species sampled in this study (*E. sinica* and *E. trifurca*) and *Welwitschia* only contain one copy of ACCD (fig. 3). The nr-ACCD of both *Sciadopitys* and gnetophytes contain 24–51 aa upstream of the usual start codon (fig. 3*a*), which were predicted to be leader sequences that target plastids (supplementary table S5, Supplementary Material online). The nr-ACCD1 of *Gnetum* was also predicted to target mitochondria, albeit with lower scores. Because the identified nr-ACCD transcript of *Welwitschia* only contains partial TP, its localization target was not clearly predicted. TPs in gnetophytes were estimated to be 29 aa (nr-ACCD2 of *Gnetum*) to 50 aa (*Ephedra*) in length. Pairwise sequence identity of orthologous TPs is >90% among congeneric species. In contrast, the predicted TPs of nr-ACCD1 and nr-ACCD2 of *Gnetum* spp. share low sequence similarity (15.6–18.2%).

A phylogenetic tree inferred from 66 ACCD sequences places the newly identified nr-ACCD of *Sciadopitys* within the cupressophytes (fig. 3*b*), consistent with the species tree. Using Tajima’s relative rate test, we found that the ACCD sequences of cupressophytes evolve faster than those of other gymnosperms except *Gnetum* (supplementary table S6, Supplementary Material online)*.* The two nr-ACCD sequences from each of the three studied *Gnetum* species form a clade with nr-ACCD sequences of *Ephedra* and *Welwitschia* (fig. 3*b*). However, the two clades of *Gnetum* nr-ACCD were not sister to each other. The nr-ACCD2 clade of the three *Gnetum* species was placed as the outgroup to gnetophytes’ nr-ACCD1 clade (fig. 3*b*), suggesting that the common ancestor of gnetophytes had two nr-ACCDs.

**Fig. 4.**
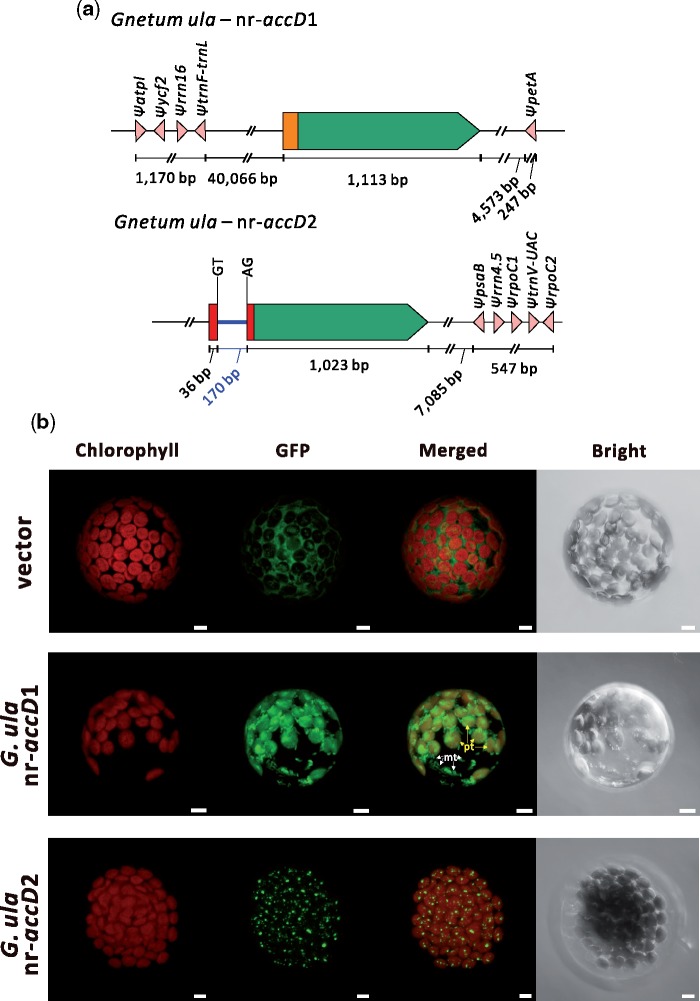
—Two nr-*accD* genes in *Gnetum ula* have distinct architectures and targeting sites. (*a*) The nr-*accD* gene architectures in the *G. ula* draft genome. The green box and blue line denote the exon and intron, respectively. Orange and red boxes correspond to the distinct TPs of the two nr-*accD*s in *G. ula*. Sequences with high similarity to *G. ula* plastid genes are designated as pink triangles. The intron donor and splice sites are labeled with GT and AG, respectively. Figures are not drawn to scale. (*b*) Transient expression assays using nr-*accD* TPs of *G. ula* indicate that the nr-*accD*1 dually targets plastids and putative mitochondria, but nr-*accD*2 likely targets plastoglobuli. Red and green signals in the first two columns represent chlorophyll autofluorescence and GFP, respectively. “Merged” column shows combined chlorophyll and GFP signals, whereas “bright” indicates bright field picture of the protoplasts. The yellow and white arrows in *G. ula* nr-*accD*1 merged column indicate plastids and mitochondria localization, respectively. Scale bar = 5 µm. Pt, plastid; mt, mitochondria.

By mapping the two nr-ACCD transcripts of *G. ula* to its draft genome, we discovered that the gene architectures of nr-*accD*1 and nr-*accD*2 are distinct (fig. 4*a*). The former contains a single exon of 1,113 bp, whereas the latter contains two exons (36 and 1,023 bp) and one intron (170 bp) with a total length of 1,229 bp (fig. 4*a*). In the nr-*accD*1 scaffold, two short fragments (1,170 and 247 bp) reside at 40-kb upstream and 4.5-kb downstream of the coding region, respectively, were identified as pseudogenes of plastome origins. Similarly, in nr-*accD*2 scaffold, a 547-bp fragment at 7-kb downstream of the stop codon is composed of five plastid pseudogenes (fig. 4*a*). None of these nuclear plastid sequences (NUPTs) contain full-length and/or in-frame coding sequences. Despite their high sequence similarities to their corresponding genes in the plastome (>85% identity), these NUPTs (33–284 bp) are not syntenic with the *G. ula* plastome (Hsu, Wu, Surveswaran, et al. 2016), implying that they were rearranged either during transfer or subsequently (Hazkani-Covo and Martin 2017).

Our first attempt to perform transient expression using the length of predicted TPs (52–60 aa) failed to detect the plastid localization in either *Gnetum* or *Sciadopitys*. By increasing the length of cloned TPs to >80 aa (extending further into the protein sequence), we concluded that the two *accD*s of *Gnetum* target distinct compartments. The TP of nr-ACCD1 dually targets plastid stroma and mitochondria, whereas TP of nr-ACCD2 potentially targets plastoglobuli, a microcompartment within the plastids (fig. 4*b*). Meanwhile, the nr-ACCD of *Sciadopitys* mainly targets cytosol with some putative plastid localization (supplementary fig. S5, Supplementary Material online). Overall, we experimentally demonstrated that the nr-ACCDs of gnetophytes and *Sciadopitys* have made up for the loss of *accD* from the plastomes of both lineages.

### Nuclear Genes Encoding Two Heteromeric ACCase Subunits (*accA* and *accB*) Are Duplicated in Various Lineages of Gymnosperms

**Fig. 5.**
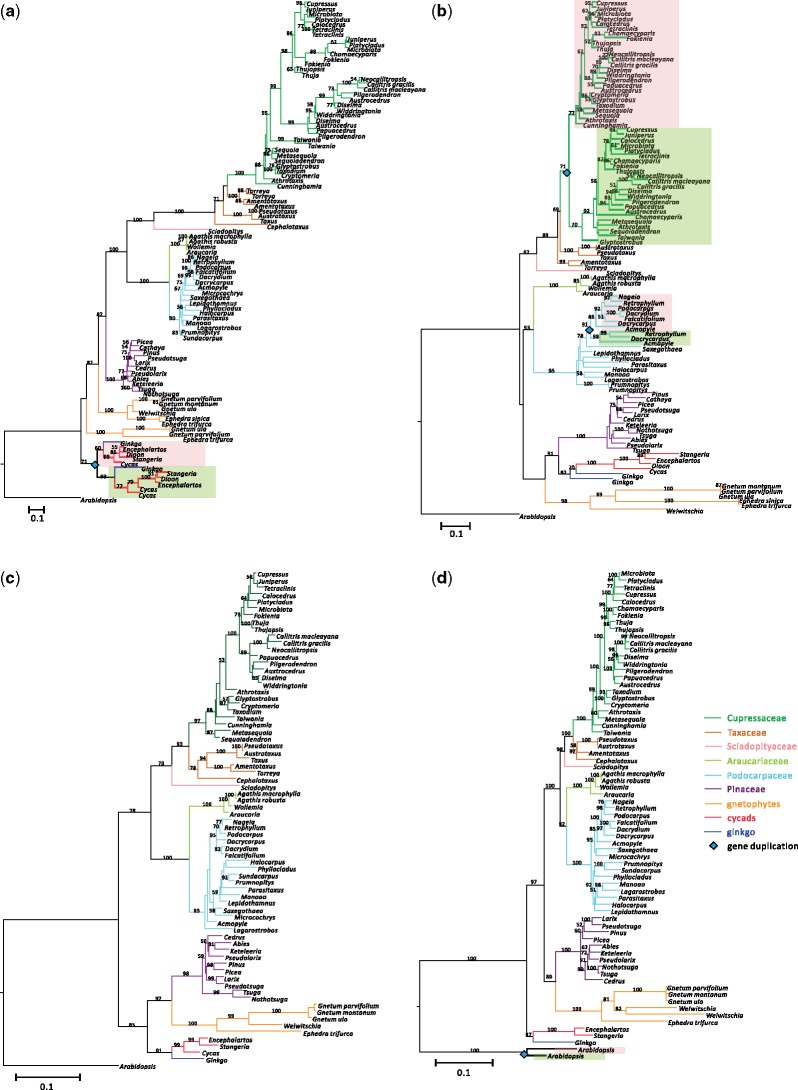
—Phylogeny-based scenario of nr-ACCase gene duplications in gymnosperms. ML trees of the four nr-ACCase proteins in gymnosperms. Numbers on the branches are bootstrap values (only >50% are shown). A blue diamond at the nodes indicates where a gene duplication event has occurred. The two duplicated gene copies are highlighted with pink and olive color shadings. The gene trees of heteromeric ACCase gene complex include (*a*) *accA*, (*b*) *accB*, and (*c*) *accC*. The homomeric ACCase (*ACC*) gene tree is depicted in (*d*).

Figure 5*a* suggests *accA* duplication occurred in the common ancestor of ginkgo and cycads. Although some gnetophyte and cupressophyte genera have at least two copies of *accA* (figs. 1 and 5*a*), we could not infer the time of their duplications in the gene tree. In the *accB* phylogeny (fig. 5*b*), however, there are two likely scenarios of gene duplication at the nodes leading to some Podocarpaceous genera and Cupressaceae. We propose that independent duplications have taken place in Cupressaceae and Podocarpaceae, as most genera of the former and some of the latter family contain two *accB* copies (fig. 5*b*). By mapping the *accA* and *accB* transcripts of *Ginkgo* and *Gnetum* to their respective genomes, we confirmed that *Ginkgo* has two copies of both *accA* and *accB*, whereas *Gnetum* has two copies of *accA* that are located in different scaffolds (supplementary fig. S6, Supplementary Material online). In contrast, single copies of heteromeric *accC* and homomeric *ACC* were found in 76 sampled species (fig. 5*c* and *d*). The number of exons in each gene (*accA–C* and *ACC*) is identical in *Ginkgo*, *Gnetum*, and *Pinus*, except for *accC*. The *accC* in *Ginkgo* has one more exon than in *Gnetum* and *Pinus* (supplementary fig. S6, Supplementary Material online)*.* However, intron lengths are highly variable between species, ranging from 75 bp to >300 kb with *Gnetum* having shorter introns. The *accA*1 of *Ginkgo* and the *accB* of *Pinus* contain extremely long introns of 59,205 and 311,403 bp, respectively (supplementary fig. S6, Supplementary Material online). The extremely long intron in the *accB* of *Pinus* is close to the longest intron (318,524 bp) ever identified in *Pinus taeda*, the genome of which contains 108 introns with length of >100 kb (Wegrzyn et al. 2014).

### Nucleotide Substitution Rates of Plastid *accD* and nr-Heteromeric ACCase Are Not Coelevated

**Fig. 6.**
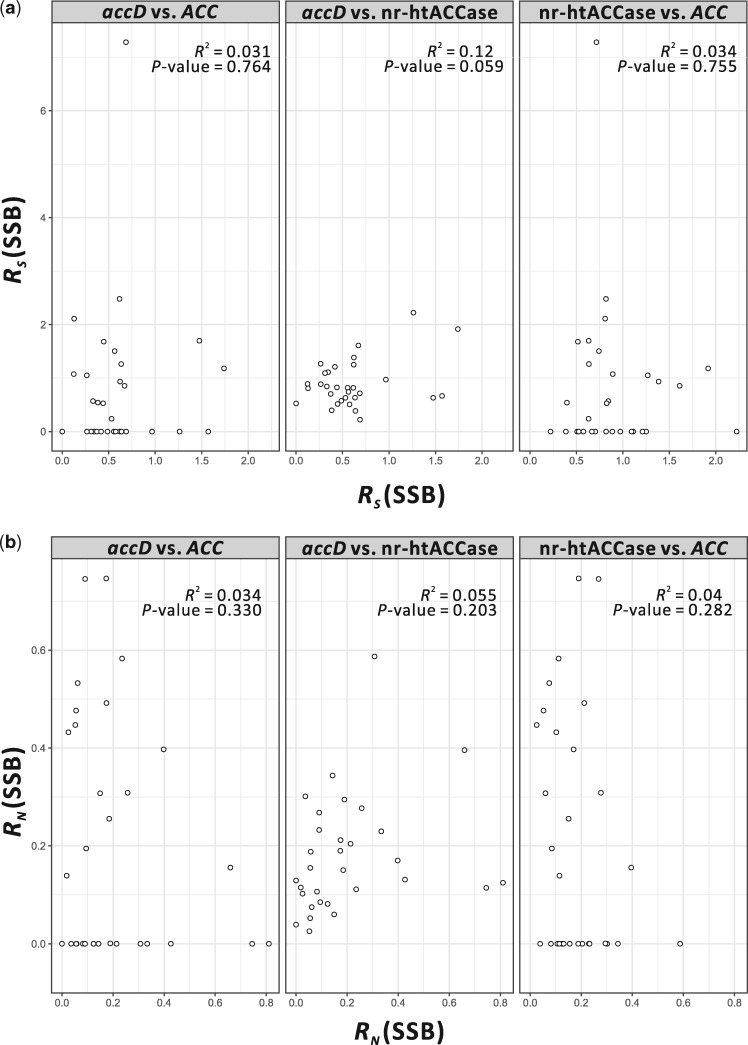
—Comparison of absolute nucleotide substitution rates between *accD*, nr-htACCase, and *ACC* of gymnosperms, except gnetophytes and *Sciadopitys*. Scatterplot and regression analyses of *accD* versus *ACC*, *accD* versus nr-htACCase, and nr-htACCase versus *ACC* for (*a*) absolute synonymous rates (*R*_S_) and (*b*) absolute nonsynonymous rates (*R*_N_), respectively. The points in each plot represent the 31 gymnosperm genera included in this analysis. Nr-htACCase, nuclear-encoded heteromeric ACCase; SSB, substitutions per site per billion years.

We calculated the nucleotide substitution rates of ACCase genes in the five major gymnosperm groups (cycads, ginkgo, pines, cupressophytes, and gnetophytes). Gnetophytes and *Sciadopitys* were excluded from our further analyses as their plastid *accD* genes were transferred to the nucleus and their nr-*accD*s have much higher substitution rates than others (supplementary fig. S7, Supplementary Material online). We only found weak correlation (*R*^2^ = 0.12, *P *=* *0.059) between the *R*_S_ of plastid *accD* and the transcripts encoding nr-heteromeric ACCase, but not *R*_N_ (*R*^2^ = 0.055, *P *=* *0.203) (fig. 6). No significant correlation was observed between plastid *accD* (or nr-heteromeric ACCase) and *ACC* at both *R*_S_ (fig. 6*a*) and *R*_N_ sites (fig. 6*b*). These results indicate that in gymnosperms, mutations in the plastid *accD* sequences have little to no effect on their nr-heteromeric ACCase.

## Discussion

### No Homomeric ACCase Was Found in the Plastids of Any Gymnosperms

Previous studies indicate that the plastids of some angiosperms contain homomeric ACCase, either from substitutions or coexisting with the heteromeric ACCase. For instance, grasses (Poaceae) and *Silene noctiflora* have completely lost heteromeric ACCase from their plastids (supplementary fig. S1, Supplementary Material online; Konishi et al. 1996; Rockenbach et al. 2016). Meanwhile, in Brassicaceae and Geraniaceae, both ACCase forms coexist in the plastids (supplementary fig. S1, Supplementary Material online; Rousseau-Gueutin et al. 2013; Park et al. 2017). Similarly, in some algal groups, such as Prasinophyceae (green algae), haptophytes, and heterokonts (red algae), the plastid heteromeric ACCase is replaced by plastid-targeted homomeric ACCase (Huerlimann and Heimann 2013; Huerlimann et al. 2015). *AccD* is absent from the plastomes of chlamydomonadalean algae but present in their nuclear genomes (Smith et al. 2013; Smith and Lee 2014). The presence of homomeric ACCase in angiosperm plastids sometimes coincides with the loss of plastid *accD* (e.g., grasses) or *accD* elongation (e.g., Geraniaceae).

This study shows that the *accD*s of some gymnosperms have been lost (e.g., gnetophytes, *Sciadopitys*) or elongated (e.g., cupressophytes). However, transcripts encoding homomeric ACCase (*ACC*) are present as a single copy in all sampled gymnosperms (figs. 1 and 5*d*) and none of them possessed TPs (supplementary table S4, Supplementary Material online). We also demonstrate that nr-ACCDs of gnetophytes and *Sciadopitys* are transcribed (figs. 3 and 4). Prediction- and experimental-based assays (fig. 4*b* and supplementary fig. S5 and supplementary table S5, Supplementary Material online) confirmed that their encoded products can be targeted to plastids to compensate for the loss of plastid-encoded *accD* in both lineages. Thus, we found no evidence of homomeric ACCase replacing or coexisting with heteromeric ACCase in the plastids of any sampled gymnosperm.

### Insertions of TR into Plastid *accD* Do Not Affect nr-Heteromeric ACCase Evolution

We verified that *accD*s are elongated by in-frame, lineage-specific TR insertions in four of the five cupressophyte families (excluding Sciadopityaceae) (table 1 and supplementary fig. S4, Supplementary Material online), and they are 2–4-fold longer than those of cycads, ginkgo, or pines (fig. 2). Different lineages have specific TRs (table 1) that likely arose in the four cupressophyte families independently. Insertion of lineage-specific TRs in the *accD* has been reported in a number of seed plants, including *Capsicum annuum* (Jo et al. 2011), *Medicago truncatula* (Gurdon and Maliga 2014), *Tsuga chinensis* (Sudianto et al. 2016), Geraniaceae (Park et al. 2017), and *Passiflora* (Rabah et al. 2019). Thus, elongation of *accD* appears to have occurred repeatedly during seed plant evolution and coincides with elevated nucleotide substitution rates. It has been hypothesized that repetitive elements in the inserted sequences promoted *accD* sequence variability (Li et al. 2018). Length polymorphism in the *accD* is likely the result of “replication slippage” as reported in the *Oenothera* plastomes (Massouh et al. 2016). Recent finding suggests that these length variations may account for the differences in competitiveness among the four plastid genotypes of *Oenothera* (Sobanski et al. 2019).

Although ACCD subunit has been known to directly interact with the heteromeric ACCA subunit (Sasaki and Nagano 2004), we did not detect significant evidence of coevolution between the plastid and nuclear genes (fig. 6). TR insertions that elongate the *accD* of cupressophytes (and *Tsuga*) mostly occur in the middle of the sequence (supplementary fig. S4, Supplementary Material online). However, catalytic sites of ACCD, which interact with the ACCA subunit, are located in the C-terminal region (Lee et al. 2004) and highly conserved among gymnosperms (supplementary fig. S4, Supplementary Material online). Thus, TR insertions do not affect plastid-nuclear interaction in the heteromeric ACCase of gymnosperms. A similar finding was previously reported in *Silene* species, where protein structural analyses indicate that large insertions in their ACCD subunit did not involve functionally important residues in protein–protein interactions (Rockenbach et al. 2016).

### The Two nr-*accD*s of *Gnetum* Were the Product of Independent Transfers Rather than Gene Duplication

Plastid *accD* genes of gnetophytes and *Sciadopitys* have been transferred to the nucleus, as nr-*accD* transcripts from the two lineages along with their plastid-targeting TPs (fig. 3*a*) attest. Based on our dated tree (supplementary fig. S3, Supplementary Material online), the *accD* transfer in gnetophytes took place after its common ancestor split from Pinaceae (ca. 245 Ma) and before the diversification of three gnetophyte genera (ca. 145 Ma), and the transfer occurred less than 253 Ma in *Sciadopitys*. *Gnetum* has two copies of nr-*accD* (figs. 1, 3, and 4), which is so far unique among green plants. This was validated in the transcriptomes of three sampled *Gnetum* species (fig. 3) and the draft genome of *G. ula* (fig. 4). The presence of two nr-*accD*s in *Gnetum* could have been caused either by 1) two independent plastid-to-nucleus transfer events or 2) the duplication of nr-*accD* after transfer to the nuclear genome. The former scenario appears to be favored because the two nr-*accD*s are distinctive in their TPs (fig. 3*a*), gene architectures and flanking NUPTs (fig. 4*a*). We propose that, in the common ancestor of gnetophytes, two copies of *accD* were independently transferred from the plastid to the nucleus. However, we could not detect any nr-ACCD2 from the transcriptomes of *Ephedra* and *Welwitschia* (fig. 3)*.* This absence may be due to the loss of nr-*accD*2 gene from both genera or, alternatively, the lack of nr-*accD*2 gene expression in the isolated RNA. The nuclear genomes of *Ephedra* and *Welwitschia* need to be sequenced in order to confirm if the nr-*accD*2 was indeed lost from both genera.

### The Two nr-ACCDs of *Gnetum* Target Different Sites

Using protoplast transient expression assays, we verified that the two nr-*accD*s of *Gnetum* are targeted to different subplastidic structures (fig. 4*b*). The nr-*accD*1 TP directed GFP to the plastid stroma (and to a lesser extent to mitochondria), whereas nr-*accD*2 TP likely targeted GFP to the plastoglobuli. The speckled pattern we observed in the nr-*accD*2 construct (fig. 4*b*) closely resembles the phytoene synthases (PSYs) of maize and rice that are delivered to the plastoglobuli (Shumskaya et al. 2012). The distinct targeting of the two nr-*accD* genes potentially suggests neo- and sub-functionalization of nr-ACCD1 and nr-ACCD2, respectively.

We were surprised to observe that nr-*accD*1 of *Gnetum* likely targets the mitochondria. Regulation of ACCase in plant mitochondria is less well characterized than its counterparts in plastids or cytosols. ACCase is reportedly absent from eudicot mitochondria, and their malonyl-CoA is synthesized using alternative pathways (Gueguen et al. 2000). However, in grasses, malonyl-CoA is generated by a homomeric ACCase that dually targets plastids and mitochondria (Focke et al. 2003). To date, little is known about ACCase in the gymnosperm mitochondria, especially whether the gymnosperm mitochondria produce malonyl-CoA via ACCase (as in grasses) or malonyl-CoA synthetase (as in eudicots) remains unclear. Nr-*accD*1 function in the mitochondria of *Gnetum* requires further investigation, as none of the other heteromeric ACCase subunits (*accA–C*) were predicted to be localized to mitochondria (supplementary table S4, Supplementary Material online). Mitochondrial localization might also just be a case of evolutionary noise in subcellular targeting evolution (Martin 2010).

Similarly, it is unusual that nr-*accD*2 targets putative plastoglobuli. Plastoglobuli are plastid lipid microcompartments that aid in plastid metabolism (reviewed in Van Wijk and Kessler [2017]). A number of nr-proteins have been reported to target plastoglobuli (Shumskaya et al. 2012; Delfosse et al. 2015). Although plastoglobuli play important roles in lipid storage and metabolism (Bréhélin and Kessler 2008), no ACCase metabolic pathway has been elucidated in the microcompartment. The only fatty acid–related enzyme identified in plastoglobuli so far is phytol ester synthase (PES; Van Wijk and Kessler 2017).

In summary, our study sheds light on the ACCase complex and its evolutionary history in seed plants. We found little evidence for coevolution between *accD* and its counterparts in heteromeric ACCase and the possible effects of TR insertions on its enzymatic function remain elusive. To date, we still rely on the bacterial ACCase structure to interpret the function and interaction of plants’ heteromeric ACCase subunits. Elucidation of the plant-specific heteromeric ACCase structure will be critical to decipher the plastid-nuclear subunit interactions in heteromeric ACCase. Moreover, further studies on the functions of the two *accD*s in *Gnetum* will be necessary to identify why the genus has two copies of the gene.

## Supplementary Material

Supplementary data are available at *Genome Biology and Evolution* online.

## Supplementary Material

Supplementary_Material_evz059

## Notes

**Data deposition:** The newly identified acetyl-CoA carboxylase genes of *Gnetum ula* were deposited at DDBJ under the accession numbers LC425655–LC425656 and LC428529–LC428533.
