# Supplementary material for: Two Independent Plastid accD Transfers to the Nuclear Genome of Gnetum and Other Insights on Acetyl-CoA Carboxylase Evolution in Gymnosperms
Source: Genome Biol Evol. 2019 Mar 29;11(6):1691–705. doi: 10.1093/gbe/evz059 (PMC6595918; doi:10.1093/gbe/evz059)
Supplement: Supplementary_Material_evz059 [file supplementary_material_evz059.pdf]

Figure S1. Modes of malonyl-CoA biosynthesis in plants. (a) Typical mode in green plants, with homomeric and heteromeric ACCase expressed at cytosol and plastids, respectively. (b) The second copy of homomeric ACCase replaces heteromeric ACCase in plastids. Some example include grasses and *Silene noctiflora*. (c) The second copy of homomeric ACCase co-exist with heteromeric ACCase in plastids. Brassicaceae and Geraniaceae plastids contain both ACCase forms. HoACCase, homomeric acetyl-CoA carboxylase; HtACCase, heteromeric acetyl-CoA carboxylase.

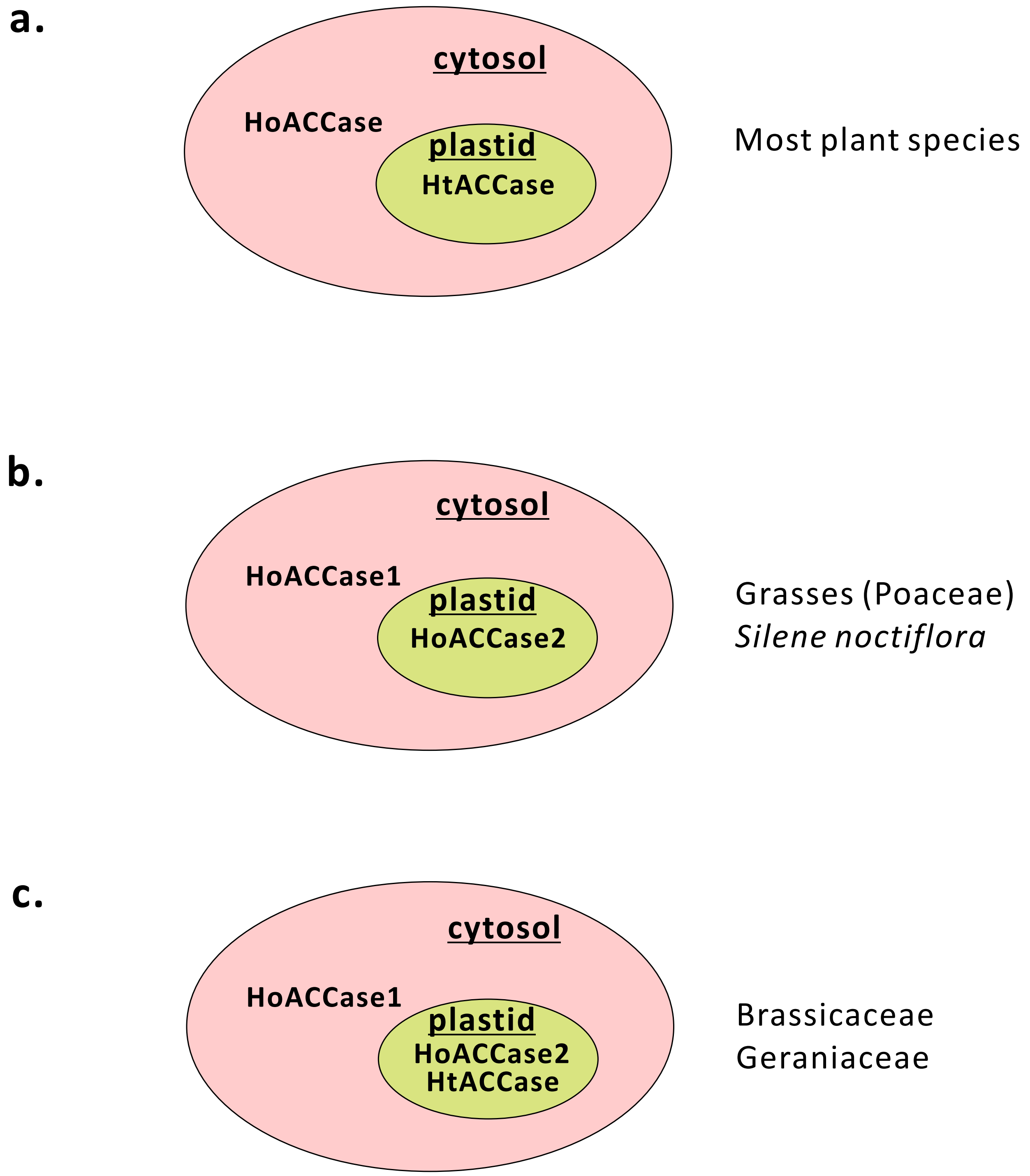

Figure S2. Constraint tree used for nucleotide substitution rates analyses.

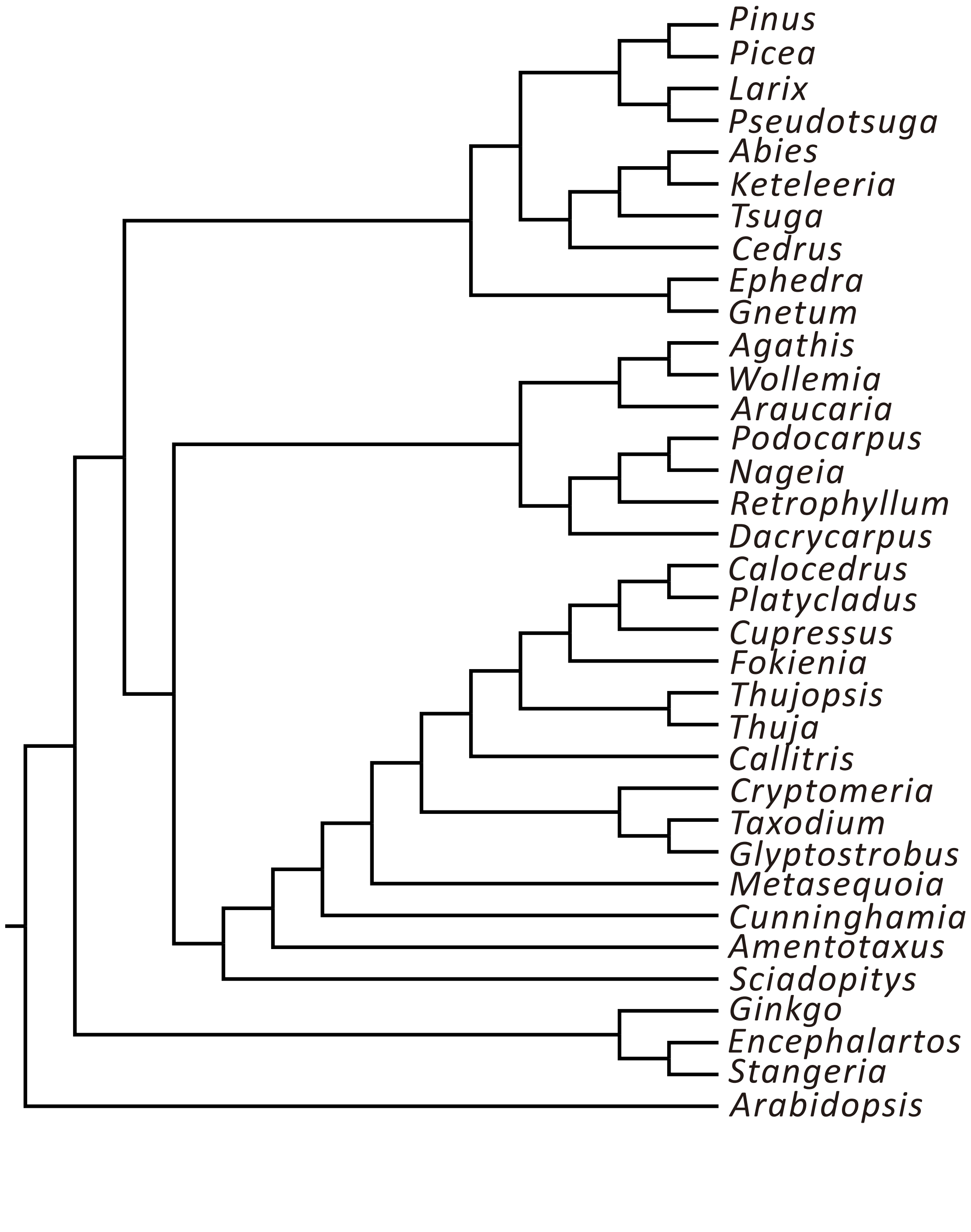

Figure S3. Estimated relative divergence times of 34 gymnosperms. A–G indicate calibration points used in this study. The 95% confidence interval of the estimated age are given for each node. MYA, million years ago

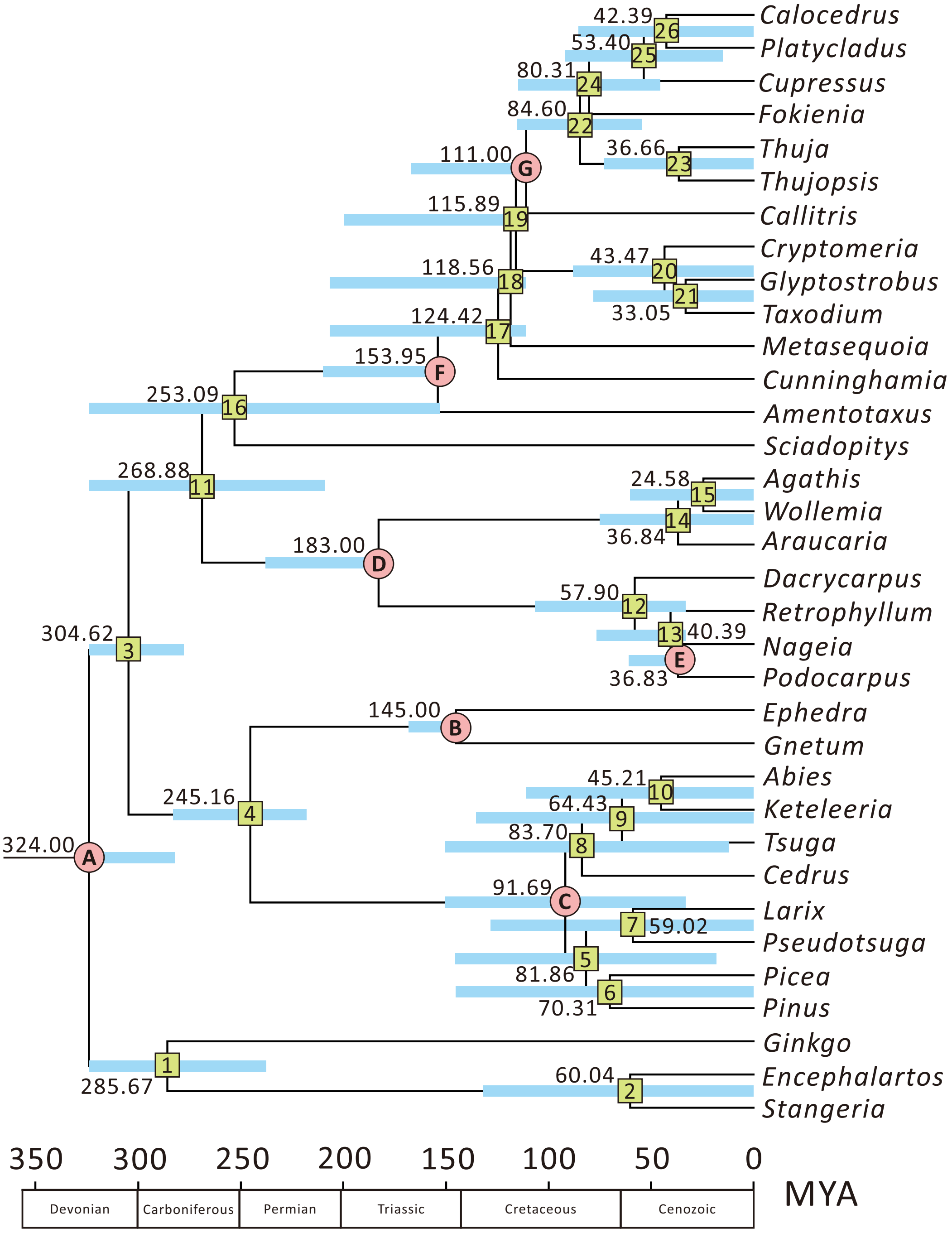

Figure S4. Alignment of pt-ACCDs from cycads, ginkgo, Pinaceae, and cupressophytes. The orange box denotes the indels specific to the ACCD of cupressophytes.

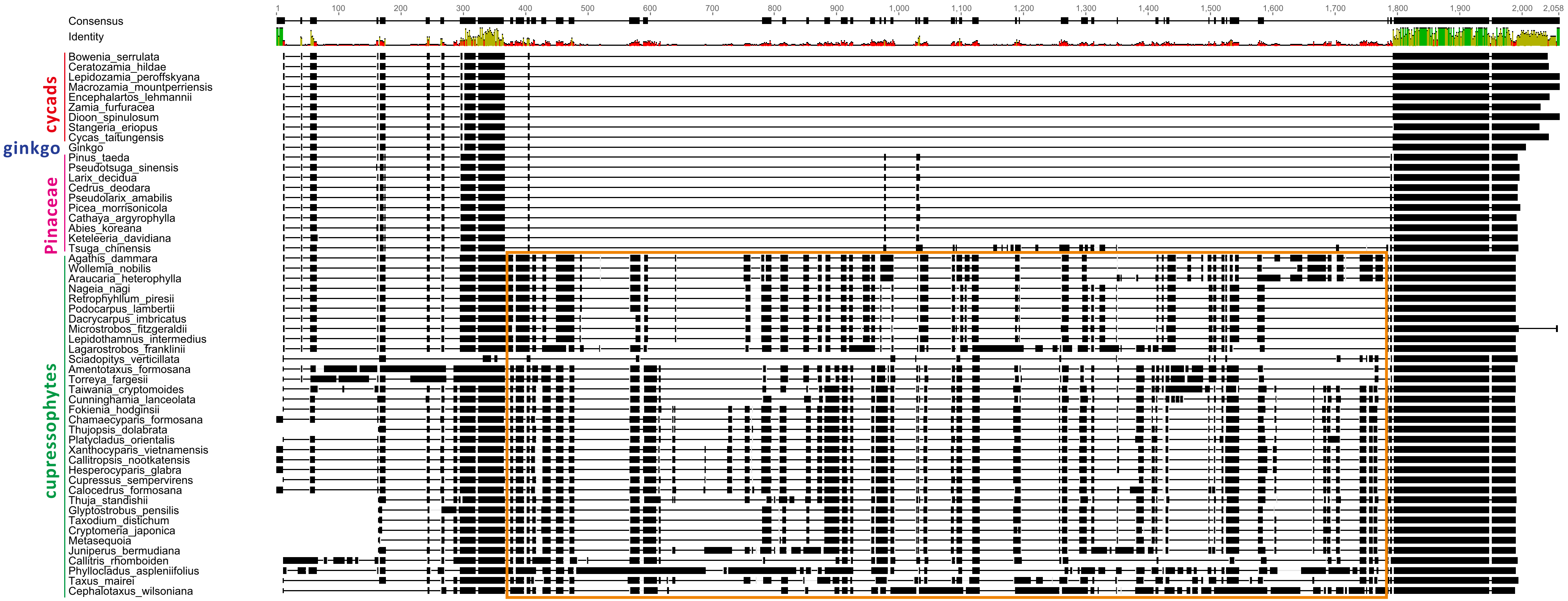

Figure S5. Transient expression assays of *Sciadopitys verticillata*'s nr-*accD*. Red and green signals in the first two columns represent chlorophyll autofluoresence and GFP, respectively. "Merged" column shows combined chlorophyll and GFP signals, while "bright" indicates bright field picture of the protoplasts. Scale bar = 5 μm.

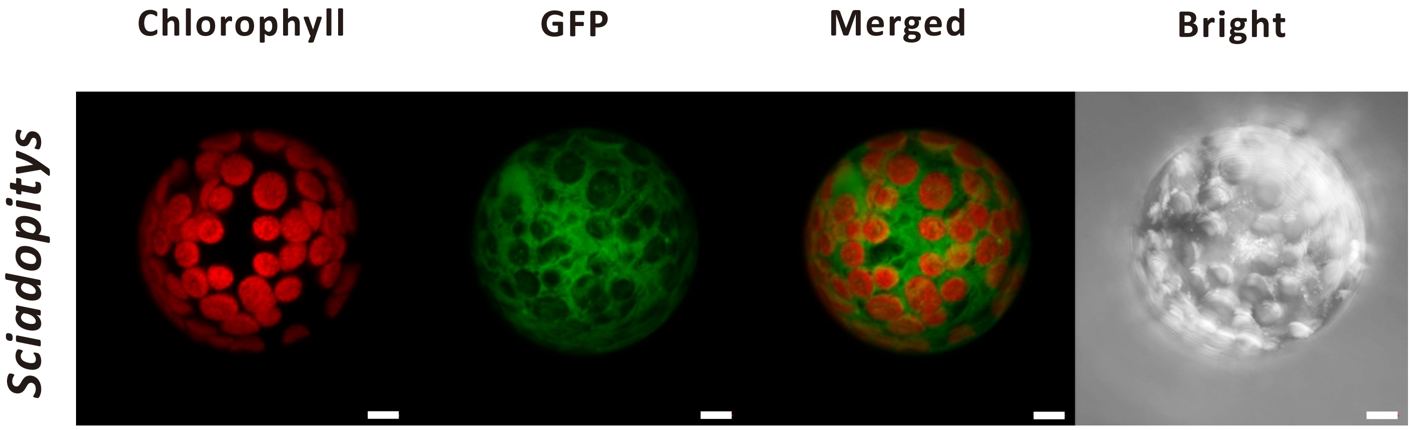

Figure S6. Comparative gene architectures of the four nr-ACCase genes in *Ginkgo, Pinus,* and *Gnetum.* Exons and introns are depicted as green boxes and black lines, respectively. The number to the right of the gene structures denote the number of exons. Lines between the genes indicate sequence similarities. Blue lines indicate that sequences are similar in the same directions, while red lines are similar in reverse directions. Color schemes at the right bottom corner depict 50% to 100% sequence similarities. We compared three genes encoding heteromeric ACCase: (a) *accA,* (b) *accB,* and (c) *accC,* and one gene encoding homomeric ACCase: (d) *ACC*.

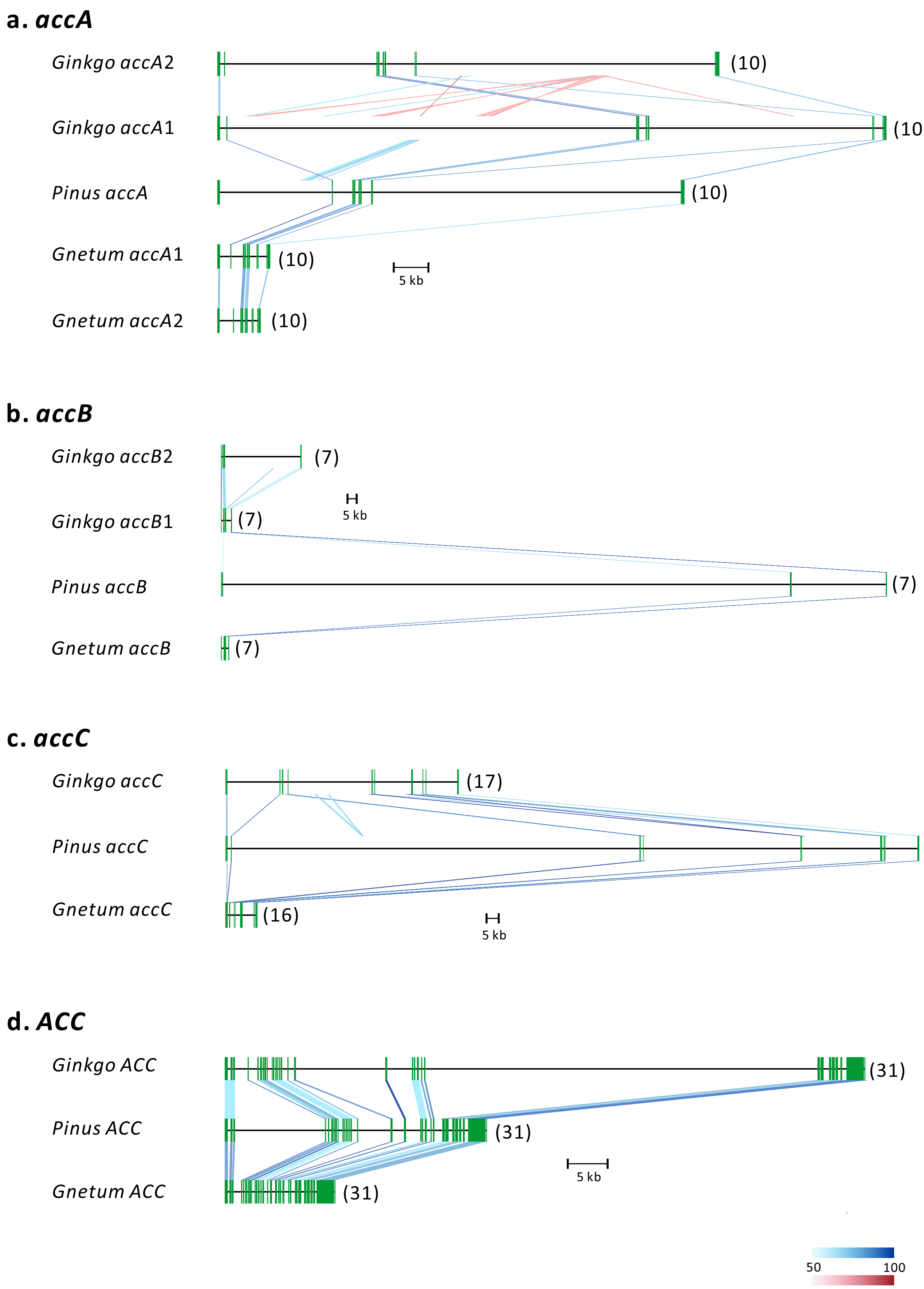

Figure S7. Comparison of absolute nucleotide substitution rates between *accD,* nr-heteromeric ACCase, and *ACC* genes of all gymnosperms. The green, blue, and red dots denote the gnetophytes, *Sciadopitys*, and other gymnosperms, respectively. nr-htACCase, nuclear-encoded heteromeric ACCase; SSB, substitutions per site per billion years.

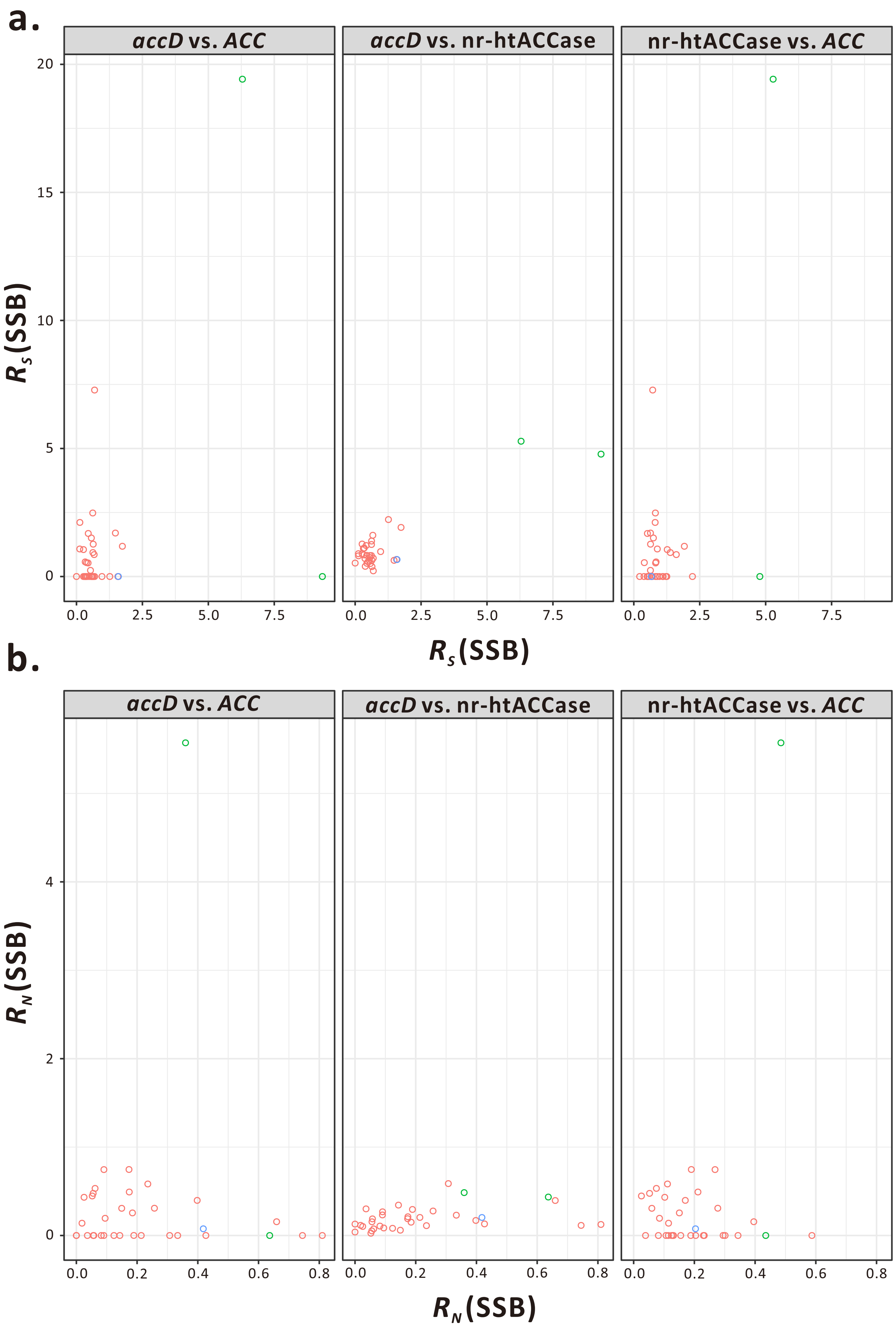

Table S1. Summary of the full-length plastid ACCD protein quantified length from 116 species of gymnosperms.

Order	Family	Genus	Species	NCBI Accession No.[a]	ACCD protein length (aa)
**Araucariales**	Araucariaceae	*Agathis*	*Agathis dammara*	AB830884	820
		Araucaria	*Araucaria cunninghamii*	KT030797	850
		Wollemia	*Wollemia nobilis*	NC_027235	800
	Podocarpaceae	*Afrocarpus*	*Afrocarpus gracilior*	AP018899	683
		Dacrycarpus	*Dacrycarpus imbricatus*	LC177621	673
		Dacrydium	*Dacrydium cupressinum*	AP018900	674
		Falcatifolium	*Falcatifolium taxoides*	**Transcriptome**	674
		Lagarostrobos	*Lagarostrobos franklinii*	AP018901	857
		Lepidothamnus	*Lepidothamnus intermedius*	AP018902	674
		Microstrobos	*Microstrobos fitzgeraldii*	AP018903	678
		Nageia	*Nageia fleuryi*	KT030795	684
			Nageia nagi	AB830885	684
			Nageia nagi	KT030794	684
		Phyllocladus	*Phyllocladus aspleniifolius*	AP018904	1173
		Podocarpus	*Podocarpus lambertii*	NC_023805	684
			Podocarpus macrophyllus	KT030792	694
			Podocarpus nerifolius	KT030793	694
			Podocarpus totara	NC_020361	684
		Prumnopitys	*Prumnopitys andina*	AP018905	683
		Retrophyllum	*Retrophyllum piresii*	KJ617081	684
		Saxegothaea	*Saxegothaea conspicua*	AP018906	655
**Cupressales**	Cupressaceae	*Athrotaxis*	*Athrotaxis cupressoides*	**Transcriptome**	689
		Austrocedrus	*Austrocedrus chilensis*	**Transcriptome**	920
		Callitris	*Callitris rhomboidea*	LC177555	720
		Callitropsis	*Callitropsis nootkatensis*	KP099642	781
			Callitropsis vietnamensis	KP099645	781
		Calocedrus	*Calocedrus formosana*	AB831010	801
			Calocedrus macrolepis	KX832621	837
		Chamaecyparis	*Chamaecyparis lawsoniana*	KX832622	764
		Cryptomeria	*Cryptomeria japonica Yaku-Sugi*	AP010967	701
			Cryptomeria japonica	AP009377	701
		Cunninghamia	*Cunninghamia lanceolata*	KC427270	741
		Cupressus	*Cupressus gigantea*	NC_028155	760
			Cupressus sempervirens	KP099643	760
		Diselma	*Diselma archeri*	**Transcriptome**	970
		Fokienia	*Fokienia hidginsii*	KX832623	746
		Glyptostrobus	*Glyptostrobus pensilis*	NC_031354	713
		Hesperocyparis	*Hesperocyparis glabra*	KP099644	781
		Juniperus	*Juniperus bermudiana*	NC_024021	900
			Juniperus cedrus	NC_028190	925

			Juniperus monosperma	NC_024022	897
			Juniperus scopulorum	NC_024023	885
			Juniperus virginiana	NC_024024	903
		Metasequoia	*Metasequoia glyptostroboides*	KR061358	697
			Metasequoia glyptostroboides	KT030796	697
		Microbiota	*Microbiota decussata*	**Transcriptome**	752
		Platycladus	*Platycladus orientalis*	KX832626	781
		Sequoia	*Sequoia sempervirens*	NC_030372	854
		Taiwania	*Taiwania cryptomoides*	AP012266	801
			Taiwania flousiana	NC_021441	796
		Taxodium	*Taxodium distichum*	LC177556	700
		Thuja	*Thuja standishii*	KX832627	748
		Thujopsis	*Thujopsis dolabrata*	KX832628	750
		Widdringtonia	*Widdringtonia cedarbengensis*	**Transcriptome**	504
	Taxaceae	*Amentotaxus*	*Amentotaxus formosana*	AP014574	911
			Amentotaxus argotaenia	KT030787	917
		Cephalotaxus	*Cephalotaxus fortunei*	KT030791	1039
			Cephalotaxus hainanensis	KT030790	1071
			Cephalotaxus oliverii	NC_021110	937
			Cephalotaxus sinensis	KT030789	1039
			Cephalotaxus wilsoniana	AP012265	1057
		Pseudotaxus	*Pseudotaxus chienii*	KT030788	658
		Taxus	*Taxus cuspidata*	KT030781	760
			Taxus mairei	KJ123824	774
			Taxus wallchiana var. chinensis	KT030782	760
			Taxus wallchiana var. chinensis	KX431996	736
			Taxus wallchiana	KT030784	737
			Taxus wallchiana var. wallchiana	KT030783	768
			Taxus x *media*	KT030780	754
		Torreya	*Torreya fargesii*	KT030785	872
			Torreya grandis	KT030786	880
**Pinales**	Pinaceae	*Abies*	*Abies koreana*	NC_026892	323
			Abies nephrolepis	KT834974	323
		Cathaya	*Cathaya argyrophylla*	AB547400	321
		Cedrus	*Cedrus deodara*	AB480043	323
		Keteleeria	*Keteleeria davidiana*	AP010820	324
		Larix	*Larix decidua*	AB501189	325
			Larix occidentalis	FJ899578	325
		Nothotsuga	*Nothotsuga longibracteata*	MG602009	309
		Picea	*Picea abies*	HF937082	327

			Picea glauca	NC_028594	327
			Picea jezoensis	NC_029374	325
			Picea morrisonicola	AB480556	327
			Picea sitchensis	KU215903	328
		Pinus	*Pinus armandii*	NC_029847	321
			Pinus bungeana	NC_028421	321
			Pinus contorta	EU998740	322
			Pinus fenzeliana var. dabenshanensis	KX255674	321
			Pinus gerardiana	EU998741	321
			Pinus koraiensis	NC_004677	321
			Pinus krempfii	EU998742	321
			Pinus labuliformis	NC_028531	322
			Pinus lambertiana	EU998743	321
			Pinus massoniana	KC427272	322
			Pinus nelsonii	EU998746	321
			Pinus sibirica	NC_028552	321
			Pinus strobus	NC_026302	321
			Pinus taeda	KC427273	322
			Pinus taiwanensis	NC_027415	322
			Pinus thunbergii	NC_001631	322
		Pseudolarix	*Pseudolarix amabilis*	NC_030631	323
		Pseudotsuga	*Pseudotsuga sinensis var. wilsoniana*	AB601120	323
		Tsuga	*Tsuga chinensis*	NC_030630	419
**Cycadales**	Cycadaceae	*Cycas*	*Cycas taitungensis*	AP009339	359
			Cycas debaoensis	KU743927	359
			Cycas revoluta	JN867588	359
			Cycas panzhihuaensis	KX713899	359
	Stangeriaceae	*Bowenia*	*Bowenia serrulata*	JX402774	358
		Stangeria	*Stangeria eriopus*	LC049067	344
	Zamiaceae	*Ceratozamia*	*Ceratozamia hildae*	LC049068	359
		Dioon	*Dioon spinulosum*	LC049070	377
		Encephalartos	*Encephalartos lehmanii*	LC049336	361
		Lepidozamia	*Lepidozamia peroffskyana*	LC049207	377
		Macrozamia	*Macrozamia mountperriensis*	LC049069	377
		Zamia	*Zamia furfuracea*	LC040885	346
**Ginkgoales**	Ginkgoaceae	*Ginkgo*	*Ginkgo biloba*	KP099648	323

[a]Transcriptome means the full length plastid ACCD proteins were derived from transcriptome assemblies

Table S2. List of transcriptomes used in this study.

Order	Family	Genus	Species	Source	Reference code
**Cupressales**	Cupressaceae	*Athrotaxis*	*Athrotaxis cupressoides*	oneKP	XIRK
		Austrocedrus	*Austrocedrus chilensis*	oneKP	YYPE
		Callitris	*Callitris gracilis*	oneKP	IFLI
			Callitris macleayana	oneKP	RMMV
		Calocedrus	*Calocedrus decurrens*	oneKP	FRPM
		Chamaecyparis	*Chamaecyparis lawsoniana*	oneKP	AIGO
		Cryptomeria	*Cryptomeria japonica*	oneKP	GMHZ
		Cunninghamia	*Cunninghamia lanceolata*	oneKP	ZQVF
		Cupressus	*Cupressus dupreziana*	oneKP	QNGJ
		Diselma	*Diselma archeri*	oneKP	GKCZ
		Fokienia	*Fokienia hodginsii*	oneKP	UEVI
		Glyptostrobus	*Glyptostrobus pensilis*	oneKP	OXGJ
		Juniperus	*Juniperus scopulorum*	oneKP	XMGP
		Metasequoia	*Metasequoia glyptostroboides*	oneKP	NRXL
		Microbiota	*Microbiota decussata*	oneKP	XQSG
		Neocallitropsis	*Neocallitropsis pancheri*	oneKP	JDQB
		Papuacedrus	*Papuacedrus papuana*	oneKP	OVIJ
		Pilgerodendron	*Pilgerodendron uviferum*	oneKP	ETCJ
		Platycladus	*Platycladus orientalis*	oneKP	BUWV
		Sequoia	*Sequoia sempervirens*	oneKP	HBGV
		Sequoiadendron	*Sequoiadendron giganteum*	oneKP	QFAE
		Taiwania	*Taiwania cryptomerioides*	oneKP	QSNJ
		Taxodium	*Taxodium distichum*	oneKP	FHST
		Tetraclinis	*Tetraclinis* sp.	oneKP	CGDN
		Thuja	*Thuja plicata*	oneKP	VFYZ
		Thujopsis	*Thujopsis dolabarata*	oneKP	NKIN
		Widdringtonia	*Widdringtonia cedarbengensis*	oneKP	AUDE
	Taxaceae	*Amentotaxus*	*Amentotaxus argotaenia*	oneKP	IAJW
		Austrotaxus	*Austrotaxus spicata*	oneKP	BTTS
		Cephalotaxus	*Cephalotaxus harringtonia*	oneKP	NVGZ
		Pseudotaxus	*Pseudotaxus chienii*	oneKP	YLPM
		Taxus	*Taxus baccata*	oneKP	WWSS
		Torreya	*Torreya nucifera*	oneKP	HQOM
	Sciadopityaceae	*Sciadopitys*	*Sciadopitys*	oneKP	YFZK
**Araucariales**	Araucariaceae	*Agathis*	*Agathis macrophylla*	oneKP	MIXZ
		Araucaria	*Araucaria rulei*	oneKP	XTZO
		Wollemia	*Wollemia nobilis*	oneKP	RSCE
	Podocarpaceae	*Acmopyle*	*Acmopyle pancheri*	oneKP	HILW
		Dacrycarpus	*Dacrycarpus compactus*	oneKP	FMWZ
		Dacrydium	*Dacrydium balansae*	oneKP	IZGN
		Falcatifolium	*Falcatifolium taxoides*	oneKP	PLYX

		Halocarpus	*Halocarpus bidwillii*	oneKP	OWFC
		Lagarostrobos	*Lagarostrobos franklinii*	oneKP	ZQWM
		Lepidothamnus	*Lepidothamnus sp.*	oneKP	BBDD
		Manoao	*Manoao colensoi*	oneKP	CDFR
		Microcachrys	*Microcachrys tetragona*	oneKP	MHGD
		Nageia	*Nageia nagi*	oneKP	UUJS
		Parasitaxus	*Parasitaxus usta*	oneKP	JZVE
		Phyllocladus	*Phyllocladus hypophyllus*	oneKP	JRNA
		Podocarpus	*Podocarpus coriaceus*	oneKP	SCEB
		Prumnopitys	*Prumnopitys andina*	oneKP	EGLZ
		Retrophyllum	*Retrophyllum minus*	oneKP	VGSX
		Saxegothaea	*Saxegothaea conspicua*	oneKP	QCGM
		Sundacarpus	*Sundacarpus amarus*	oneKP	KLGF
**Gnetales**	Gnetaceae	*Gnetum*	*Gnetum montanum*	oneKP	GTHK
			Gnetum parvifolium	NCBI SRA	SRX1133345
			Gnetum ula	This study	-
**Welwitschiales**	Welwitschiaceae	*Welwitschia*	*Welwitschia mirabilis*	oneKP	TOXE
**Ephedrales**	Ephedraceae	*Ephedra*	*Ephedra sinica*	oneKP	VDAO
			Ephedra trifurca	NCBI TSA	GBKT01
**Pinales**	Pinaceae	*Abies*	*Abies lasiocarpa*	oneKP	VSRH
		Cathaya	*Cathaya argyrophylla*	oneKP	NPRL
		Cedrus	*Cedrus libani*	oneKP	GGEA
		Keteleeria	*Keteleeria evelyniana*	oneKP	JUWL
		Larix	*Larix speciosa*	oneKP	WVWN
		Nothotsuga	*Nothotsuga longibracteata*	oneKP	AREG
		Picea	*Picea engelmannii*	oneKP	AWQB
		Pinus	*Pinus parviflora*	oneKP	IIOL
		Pseudolarix	*Pseudolarix amabilis*	oneKP	AQFM
		Pseudotsuga	*Pseudotsuga wilsoniana*	oneKP	IOVS
		Tsuga	*Tsuga heterophylla*	oneKP	GAMH
**Cycadales**	Cycadaceae	*Cycas*	*Cycas micholitzii*	oneKP	XZUY
		Stangeria	*Stangeria eriopus*	oneKP	KAWQ
	Zamiaceae	*Dioon*	*Dioon edule*	oneKP	WLIC
		Encephalartos	*Encephalartos barteri*	oneKP	GNQG
**Ginkgoales**	Ginkgoaceae	*Ginkgo*	*Ginkgo biloba*	oneKP	SGTW

Table S3. List of primers used for cDNA amplification of the putative transit peptides from *Gnetum ula* and *Sciadopitys*.

Name	Primer sequences (5' to 3')
Gula1F	TAAACTATTCTAGATGATGGCCACCATCA
Gula1R	TAAACTATGGATCCCCAGGTCGCCCGT
Gula2F	TAAACTATTCTAGATGATGGCTTCCCTCTCTG
Gula2R	TAAACTATGGATCCCCTTGCTGAATGCA
SciF	TAAACTATTCTAGATGATGGCAGCAGCAG
SciR	TAAACTATGGATCCCAGAAGGCTCAGACCT

Table S4. Score and target of predicted transit peptide in the identified *accA–accC* and *ACC*.

Groups	Species	Localization target prediction[a]			
		accA	*accB*	*accC*	*ACC*
**Cycads**	*Cycas micholitzii*	NA/NA	C (0.649)	NA	NA
	Dioon edule	C (0.692)/NA	C (0.986)	NA	NA
	Encephalartos barteri	C (0.67)/C (0.832)	C (0.995)	C (0.999)	NA
	Stangeria eriopus	C (0.933)/NA	C (0.971)	C (0.999)	NA
**Ginkgo**	*Ginkgo biloba*	C (0.673)/C (0.865)	C (0.803)/C (0.989)	C (0.995)	NA
**Pinaceae**	*Abies lasiocarpa*	C (0.97)	C (1.0)	C (0.999)	NA
	Cathaya argyrophylla	C (0.987)	C (0.998)	C (0.991)	NA
	Cedrus libani	C (0.993)	NA	C (0.977)	NA
	Keteleeria evelyniana	C (0.997)	C (0.998)	C (0.987)	NA
	Larix speciosa	C (0.998)	C (0.998)	C (0.993)	NA
	Nothotsuga longibracteata	C (0.968)	C (0.999)	C (0.983)	NA
	Picea engelmanii	C (0.999)	C (0.998)	C (0.991)	NA
	Pinus parviflora	C (0.974)	NA	C (0.993)	NA
	Pseudolarix amabilis	C (0.998)	C (1.0)	C (0.999)	NA
	Pseudotsuga menziesii	NA	NA	C (0.99)	NA
	Tsuga heterophylla	C (0.999)	C (0.999)	C (0.941)	NA
**Gnetophytes**	*Ephedra sinica*	C (0.987)	NA	NA	NA
	Gnetum montanum	C (0.988)	NA	C (0.957)	NA
**Cupressophytes (Araucariaceae)**	*Agathis robusta*	C (0.764)	C (1.0)	C (0.999)	NA
	Araucaria rulei	C (0.951)	C (1.0)	C (0.999)	NA
	Araucaria sp.	C (0.764)	C (1.0)	C (0.999)	NA
	Wollemia nobilis	C (0.839)	C (1.0)	C (0.999)	NA
**Cupressophytes (Podocarpaceae)**	*Acmopyle pancheri*	C (0.909)	C (0.996)/C (0.992)	C (0.999)	NA
	Dacrycarpus compactus	C (0.799)	C (0.999)/C (0.998)	C (0.999)	NA
	Dacrydium balansae	C (0.826)	C (0.999)	C (0.999)	NA
	Falcatifolium taxoides	C (0.849)	C (0.999)	C (0.999)	NA
	Halocarpus bidwillii	C (0.981)	C (0.998)	C (0.999)	NA
	Lagarostrobos franklinii	NA	C (0.999)	C (0.999)	NA
	Manoao colensoi	C (0.833)	C (0.995)	C (0.999)	NA
	Microcachrys tetragona	C (0.698)	C (1.0)	C (0.999)	NA
	Microstrobos fitzgeraldii	C (0.805)	NA	C (0.999)	NA
	Nageia nagi	C (0.841)	C (1.0)	C (0.995)	NA
	Parasitaxus usta	C (0.98)	C (1.0)	C (0.997)	NA
	Phyllocladus hypophyllus	C (0.879)	C (0.999)	C (1.0)	NA
	Podocarpus coriaceus	C (0.976)	C (1.0)	C (0.999)	NA
	Prumnopitys andina	C (0.897)	C (0.996)	C (0.998)	NA
	Retrophyllum minus	C (0.873)	C (1.0)	C (0.999)	NA
	Saxegothaea conspicua	C (0.834)	C (1.0)	C (0.999)	NA
	Sundacarpus amarus	C (0.957)	NA	C (0.999)	NA

**Cupressophytes (Sciadopityaceae)**	*Sciadopitys verticillata*	M (0.868)	C (0.96)	C (0.994)	NA
**Cupressophytes (Taxaceae)**	*Amentotaxus argotaenia*	NA	NA	C (0.999)	NA
	Austrotaxus spicata	C (0.934)	C (1.0)	C (0.999)	NA
	Cephalotaxus harringtonia	NA	NA	C (0.999)	NA
	Pseudotaxus chienii	C (0.934)	C (1.0)	NA	NA
	Taxus baccata	NA	NA	C (0.998)	NA
	Torreya nucifera	NA	NA	NA	NA
**Cupressophytes (Cupressaceae)**	*Athrotaxis cupressoides*	C (0.887)	C (0.999)/C (0.723), M (0.837)	C (1.0)	NA
	Austrocedrus chilensis	C (0.988)/NA	C (0.996)/C (0.999), M (0.669)	C (1.0)	NA
	Callitris gracilis	M (0.787)	C (0.999)/C (0.99)	C (1.0)	NA
	Callitris macleayana	M (0.787)	C (0.998), M (0.637)/C (0.993), M (0.606)	C (1.0)	NA
	Calocedrus decurrens	C (0.956)	C(0.998)/C (0.997), M (0.693)	C (1.0)	NA
	Chamaecyparis lawsoniana	C (0.947)	C (0.995)/NA	C (0.999)	NA
	Cryptomeria japonica	C (0.887)	C (0.999)/M (0.996)	C (1.0)	NA
	Cunninghamia lanceolata	C (0.992)	NA	C (0.999)	NA
	Cupressus dupreziana	C (0.881)	C (0.987)/ C (0.999), M (0.809)	C (1.0)	NA
	Diselma archeri	C (0.974)/M (0.976)	C (0.999)/C (0.996)	C (1.0)	NA
	Fokienia hodginsii	C (0.935)/C (0.873)	C (0.995)/NA	C (0.999)	NA
	Glyptostrobus pensilis	C (0.735)	C (0.999)/C (0.98), M (0.966)	C (0.999)	NA
	Juniperus scopulorum	C (0.925)/C (0.961)	C (0.991)/ C (0.997), M (0.763)	C (0.999)	NA
	Metasequoia glyptostroboides	C (0.818)	C (0.999)/C (0.997)	C (0.999)	NA
	Microbiota decussata	C (0.953)/M (0.801)	C (0.996)/ C(0.981), M (0.63)	C (0.998)	NA
	Neocallitropsis pancheri	NA	C (0.999)/C (0.994), M (0.672)	C (1.0)	NA

Papuacedrus papuana	C (0.963)	C (0.999), M (0.668)/C (0.996), M (0.685)	C (1.0)	NA
Platycladus orientalis	C (0.982)/ M (0.801)	C (0.994)/ C (0.984)	C (0.997)	NA
Pilgerondendron uviferum	C (0.946)/NA	C (0.999), M (0.697)/C (0.941), M (0.721)	C (1.0)	NA
Sequoia sempervirens	M (0.913)	C (0.999)	C (1.0)	NA
Sequoiadendron giganteum	C (0.776)	C (0.996)/C (0.994)	C (0.999)	NA
Taiwania cryptomerioides	C (0.949)/C (0.963)	C (0.994)	C (0.999)	NA
Taxodium distichum	C (0.735)	NA	C (1.0)	NA
Tetraclinis sp.	C (0.937)	C (0.965)/C (0.941)	NA	NA
Thuja plicata	C (0.896)	C (0.988), M (0.645)	C (0.999)	NA
Thujopsis dolabrata	C (0.943)	C (0.997)	C (0.999)	NA
Widdringtonia cedarbergensis	C (0.982)/M (0.973)	C (0.999)/C (0.996)	C (1.0)	NA

[a]Predicted by LOCALIZER 1.0, either to chloroplast (C), mitochondria (M), or not applicable (NA). Should there be two genes in one species, the localization of both genes were predicted (copy1/copy2).

Table S5. Score and target of predicted transit peptide in the identified nr-ACCD proteins[a].

Species	TargetP	LOCALIZER	Predotar	ProteinProwler
Ephedra sinica	**C (0.739)**	**C (0.999)**	**C (0.77)**, O (0.21)	**C (0.97)**
Ephedra trifurca	**C (0.817)**	**C (1.0)**	**C (0.62)**, M (0.31), O (0.26)	**C (0.98)**
Welwitschia mirabilis	**O (0.637)**, M (0.322)	N/A	N/A	**O (0.85)**
*Gnetum ula accD*1	**C (0.870)**	**C (0.98)** M (0.824)	**C (0.95)**, M (0.64)	**C (0.94)**
*Gnetum ula accD*2	**C (0.643)**, M (0.250)	N/A	**C (0.60)**, O (0.37)	**C (0.98)**
*Gnetum parvifolium accD*1	**C (0.886)**, M (0.235)	**C (0.982)** M (0.899)	**C (0.86),** M (0.48)	**C (0.95)**
*Gnetum parvifolium accD*2	**C (0.730)**, M (0.293)	N/A	N/A	**C (0.96)**
*Gnetum montanum accD*1	**C (0.937)**, M (0.271)	**C (0.979)**, M (0.874)	**C (0.91)**, M (0.68)	**C (0.96)**
*Gnetum montanum accD*2	**C (0.588)**, M (0.245)	N/A	C (0.40), **O (0.55)**	**C (0.94)**
Sciadopitys verticillata	**C (0.835)**	**C (1.0)**	**C (0.94)**	**C (0.98)**

[a]Bold numbers indicated the location where nuclear *accD* protein are likely transported to, either chloroplast (C), mitochondria (M), others (O), or not applicable (N/A). The maximum scores from all programs is 1.0.

Table S6. Tajima's relative rate test for the amino acid alignment of *accD* genes in gymnosperms.

Sequence 1	Sequence 2	Outgroup	Tajima's relative rate test[a]
Gn. parvifolium nr-*accD*1	*Gn. parvifolium* nr-*accD*2	*Ephedra sinica*	χ^2 = 2.77, *P*-value = 0.096
Gn. montanum nr-*accD*1	*Gn. montanum* nr-*accD*2	*Ephedra sinica*	χ^2 = 0.91, *P*-value = 0.339
Gn. ula nr-*accD*1	*Gn. ula* nr-*accD*2	*Ephedra sinica*	χ^2 = 1.70, *P*-value = 0.192
Gn. parvifolium nr-*accD*1	*Gn. parvifolium* nr-*accD*2	*Marchantia polymorpha*	χ^2 = 0.24, *P*-value = 0.622
Gn. montanum nr-*accD*1	*Gn. montanum* nr-*accD*2	*Marchantia polymorpha*	χ^2 = 0.82, *P*-value = 0.366
Gn. ula nr-*accD*1	*Gn. ula* nr-*accD*2	*Marchantia polymorpha*	χ^2 = 2.77, *P*-value = 0.096
Ephedra sinica	*Gn. montanum* nr-*accD*1	*Marchantia polymorpha*	**χ^2 = 4.12, *P*-value = 0.042***
Welwitschia mirabilis	*Gn. montanum* nr-*accD*1	*Marchantia polymorpha*	**χ^2 = 4.57, *P*-value = 0.033***
Ephedra sinica	*Gn. montanum* nr-*accD*2	*Marchantia polymorpha*	χ^2 = 0.58, *P*-value = 0.446
Welwitschia mirabilis	*Gn. montanum* nr-*accD*2	*Marchantia polymorpha*	χ^2 = 1.13, *P*-value = 0.289
Juniperus bermudiana	*Ginkgo biloba*	*Marchantia polymorpha*	**χ^2 = 16.49, *P*-value < 0.001*****
Juniperus bermudiana	*Cycas taitungensis*	*Marchantia polymorpha*	**χ^2 = 15.51, *P*-value < 0.001*****
Juniperus bermudiana	*Pinus taeda*	*Marchantia polymorpha*	**χ^2 = 19.84, *P*-value < 0.001*****
Juniperus bermudiana	*Gn. montanum* nr-*accD*1	*Marchantia polymorpha*	χ^2 = 2.58, *P*-value = 0.108
Juniperus bermudiana	*Ephedra sinica*	*Marchantia polymorpha*	**χ^2 = 8.45, *P*-value = 0.003****
Juniperus bermudiana	*Welwitschia mirabilis*	*Marchantia polymorpha*	**χ^2 = 8.33, *P*-value = 0.004****
Juniperus bermudiana	*Torreya fargesii*	*Marchantia polymorpha*	χ^2 = 1.29, *P*-value = 0.257
Juniperus bermudiana	*Sciadopitys verticillata*	*Marchantia polymorpha*	χ^2 = 0.75, *P*-value = 0.385
Juniperus bermudiana	*Dacrycarpus imbricatus*	*Marchantia polymorpha*	χ^2 = 3.17, *P*-value = 0.075
Juniperus bermudiana	*Agathis dammara*	*Marchantia polymorpha*	χ^2 = 2.12, *P*-value = 0.146

[a]The asterisks indicate the *P*-value significance of < 0.05 (*), < 0.005 (**), and < 0.001 (***)
